# Interfacial Electronic Regulation in N-Doped Carbon-Encapsulated NiCo Alloy Enables High-Current Nitrate-to-Ammonia Electrosynthesis

**DOI:** 10.1007/s40820-026-02359-6

**Published:** 2026-11-09

**Authors:** Qizhi Hu, Siyu Zhu, Biao Meng, Mingdong Sun, Mingzhen Wang, Xiaoling Liu, Shibo Xi, Xiao Chi, Xiaojiang Yu, Jing Wu, Yu Zhou, Yizhong Huang, Jun Wang

**Affiliations:** 1 State Key Laboratory of Materials-Oriented Chemical Engineering, College of Chemical Engineering, Nanjing Tech University, Nanjing, 211816 People’s Republic of China; 2 School of Materials Science and Engineering, Nanyang Technological University, 50 Nanyang Avenue, Singapore, 639798 Singapore; 3 Institute of Sustainability for Chemicals, Energy and Environment (ISCE2), Agency for Science, Technology and Research (A*STAR), 1 Pesek Road Jurong Island, Singapore, 627833 Singapore; 4 Department of Physics, National University of Singapore, Singapore, 117576 Singapore; 5 Singapore Synchrotron Light Source, National University of Singapore, 5 Research Link, Singapore, 117603 Singapore

**Keywords:** Ammonia electrosynthesis, Electrochemical nitrate reduction, Alloy catalysts, Heterogeneous catalysis, Carbon materials

## Abstract

Electrochemical nitrate reduction (NO_3_^−^RR) offers a route to couple wastewater remediation with green ammonia (NH_3_) synthesis, but remains kinetically and mechanistically challenging under high-current-density operation. Herein, an N-doped carbon-encapsulated NiCo alloy catalyst (NiCo@NC) is derived from a NiCo-ZB precursor supported on conductive carbon black. The cathodic NO_3_^−^RR over NiCo@NC achieves an NH_3_ yield rate of 187 mol h^−1^ g_cat_^−1^ with 95% Faradaic efficiency (FE) at −0.6 V_RHE_ in an H-type cell and sustains ~92% FE at 2.0 A cm^−2^ under a cell voltage of 3.3 V in a membrane–electrode assembly. Pairing this cathodic NO_3_^−^RR with anodic methanol oxidation to formate enables simultaneous NH_3_ and formate production at 2.1 A cm^−2^ under a cell voltage of 2.8 V, while maintaining NH_3_ and formate FEs above 90% and 96%, respectively. *In situ* Fourier transform infrared and *operando* X-ray absorption fine structure spectra reveal preferential *NO stabilization and reversible Co valence/coordination, while theoretical calculations disclose interfacial charge polarization between NiCo alloy core and N-doped carbon shell, fabricating dual sites, comprising electron-deficient graphitic carbon and electron-rich pyridinic-N domains, that accelerate water dissociation/proton supply and lower the barrier of the rate-determining *NO → *NOH step. These insights establish alloy@NC interface regulation as a general strategy for high-rate NO_3_^−^RR.

**Supplementary Information:**

The online version contains supplementary material available at https://doi.org/10.1007/s40820-026-02359-6.

## Highlights

- N-doped carbon-encapsulated NiCo alloy enables high-rate nitrate-to-ammonia electrosynthesis.
- Alloy–carbon interfacial polarization creates dual sites for water activation and intermediate hydrogenation.
- Ampere-level NH_3_ synthesis is achieved in membrane–electrode assembly with efficient paired NH_3_/formate co-electrolysis.

**Supplementary Information:**

The online version contains supplementary material available at https://doi.org/10.1007/s40820-026-02359-6.

## Graphical abstract

**Figure:**
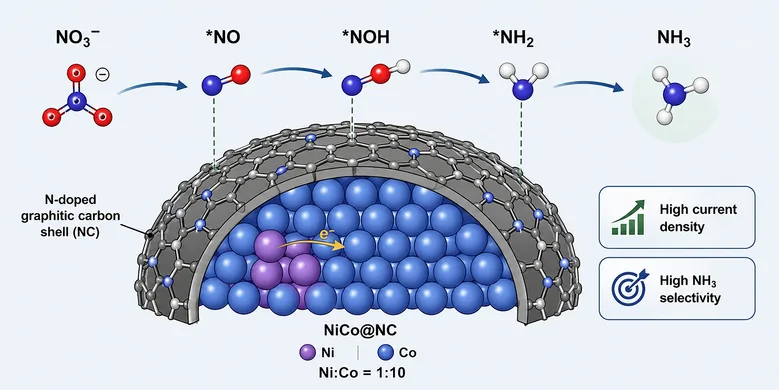

## Introduction

Electrochemical nitrate reduction reaction (NO_3_^−^RR) driven by renewable electricity is particularly attractive for green ammonia (NH_3_) synthesis because of the relatively low N=O bond dissociation energy (204 kJ mol^−1^) and the high solubility of nitrate in aqueous electrolytes [1–5]. Moreover, nitrate is a pervasive pollutant originating from agricultural runoff and industrial effluents, allowing NO_3_^−^RR to simultaneously couple nitrate remediation with NH_3_ production [6–10]. Despite these advantages, NO_3_^−^RR becomes particularly challenging under practical high-current operation, where rapid proton–electron transfer and controlled stabilization of multiple nitrogen-containing intermediates must be achieved simultaneously [11–16]. The reaction proceeds through a complex eight-electron, nine-proton cascade (NO_3_^−^ → NO_2_^−^ → NO → NH_2_OH → NH_3_), often resulting in sluggish kinetics, poor control over intermediate conversion, and severe competition from the hydrogen evolution reaction (HER) [17–19]. Consequently, NO_3_^−^RR performance is dictated by the adsorption energetics of key intermediates (*NO_3_^−^, *NO_2_^−^, *NO, *NH_x_) together with the delicate balance of hydrogen adsorption, rendering catalyst electronic structure and surface chemistry decisive factors [20–22].

To address these challenges, catalysts capable of regulating multiple elementary steps in a coordinated manner are required. Dual-active-site catalysts have therefore attracted particular interest, as they integrate complementary functionalities for nitrate activation and intermediate hydrogenation [23–25]. Conceptually, dual-site architectures can be realized in several representative forms, including bimetallic/alloy catalysts, metal–support interfacial catalysts, Me–N–C-based catalysts, and metal-free heteroatom/defect-engineered carbon catalysts [26–31]. Among these, carbon-based dual-site systems are attractive owing to their corrosion resistance, structural robustness, and tunable defect/heteroatom chemistry, yet they often suffer from limited intrinsic activity, largely owing to suboptimal electrical conductivity and sluggish charge transfer kinetics [32, 33]. An emerging strategy to overcome these limitations is to construct metal (or alloy)@carbon core–shell architectures, in which the carbon shell provides chemical protection and tunable surface motifs, while the encapsulated metal core serves as an electron reservoir to promote charge transport and interfacial electron redistribution [33–37]. In particular, integrating alloy nanoparticles (NPs) within an N-doped carbon shell combines the merits of alloying and carbon encapsulation. Such carbon-encapsulated alloy catalysts have shown compelling performance in several electrocatalytic reactions, most notably in H_2_O_2_ electrosynthesis *via* the two-electron oxygen reduction reaction (2e^−^ ORR) and in CO_2_ reduction, where metal–carbon interfacial coupling and heteroatom-doped carbon shells enable high selectivity and durability [35, 38, 39]. To the best of our knowledge, carbon-encapsulated alloy catalysts have rarely been applied to NO_3_^−^RR, leaving a key opportunity to leverage alloy-induced charge transfer and N-doped carbon shell polarization to create robust, high-rate dual active centers for nitrate activation, proton supply, and selective hydrogenation under device-relevant operation.

Herein, we report a carbon-encapsulated NiCo alloy nanocatalyst (NiCo@NC) synthesized *via* assembly of a NiCo-ZIF-BDC (NiCo-ZB) precursor on conductive carbon black followed by controlled pyrolysis. The optimized NiCo@NC catalyst delivers an outstanding NH_3_ yield rate of 187 mol h^−1^ g_cat_^−1^ with a Faradaic efficiency (FE) of 95% in an H-type cell and sustains high-current operation up to 2.0 A cm^−2^ with ~92% FE in a membrane–electrode assembly (MEA) system. Furthermore, coupling NiCo@NC-catalyzed NO_3_^−^RR at the cathode with NiCoSₓ/NF-catalyzed methanol oxidation at the anode enables simultaneous NH_3_ and formate production in a flow cell at 2.1 A cm^−2^. Mechanistic investigations reveal that the exceptional performance of NiCo@NC is closely associated with synergistic alloy–carbon interfacial regulation. Alloying induces the electron transfer from Ni to Co, generating electron-enriched Co species. Meanwhile, interfacial coupling between the N-doped carbon shell and the NiCo alloy creates charge-polarized dual sites composed of electron-rich pyridinic-N and electron-deficient graphitic carbon. The former promotes nitrate adsorption and intermediate activation, whereas the latter facilitates fast water dissociation, controlled proton supply, and selective hydrogenation. This interfacial electronic coupling underpins the high activity, selectivity, and durability of NiCo@NC under industrially relevant high-current-density conditions.

## Experimental Section

### Preparation of Active Carbon Black

Commercial superconducting carbon black (K90, 0.5 g) was dispersed in 40 wt% HNO_3_ solution (40 mL) and then refluxed at 80 °C for 24 h. After cooling to room temperature, the resulting solid was isolated by filtration, washed with water, and dried at 60 °C to provide active carbon black.

### Preparation of Metal Loaded Carbon

Active carbon black (0.2 g) was dispersed in methanol (100 mL) containing Co(NO_3_)_2_ ·6H_2_O (5 mmol) and Ni(CH_3_COO)_2_·4H_2_O (4 mmol). The resulting suspension was then combined with a mixed solution of chloroform (60 mL), N,N-dimethylformamide (60 mL), 2-methylimidazole (20 mmol), and terephthalic acid (1.5 mmol), followed by stirring at room temperature for 48 h. The obtained solid was collected by filtration, washed with methanol, and dried at 60 °C in the air for 12 h to yield the MOF precursor denoted as NiCo-ZIF-BDC (NiCo-ZB).

Subsequent pyrolysis was conducted in a tubular furnace under continuous N_2_ flow (50 mL min^−1^). The dried NiCo-ZB precursor was placed in a quartz boat, heated from room temperature to 900 °C at a ramping rate of 2 °C min^−1^, maintained at 900 °C for 2 h, and then naturally cooled to room temperature under N_2_ atmosphere. The carbonized product was designated as NiCo@NC. For comparison, Ni-ZIF-BDC (Ni-ZB) and Co-ZIF-BDC (Co-ZB) precursors were synthesized under identical conditions using Ni(CH_3_COO)_2_ (4 mmol) or Co(NO_3_)_2_·H_2_O (5 mmol) as the sole metal precursor, respectively. The corresponding pyrolyzed products, Ni@NC and Co@NC, were prepared from Ni-ZB and Co-ZB precursors via the same heat-treating protocol. NiCo@NC-NB was prepared following the same procedures of NiCo@NC without added active carbon black in the synthesis of the NiCo-ZB precursor.

### Electrochemical Measurements

The NO_3_^−^RR performance was evaluated on a CHI760E electrochemical workstation (Shanghai Chenhua, China) using a conventional H-type electrolytic cell, where the cathodic and anodic chambers were separated by a Nafion 117 membrane. A Hg/HgO electrode and a Pt foil were employed as the reference and counter electrodes, respectively, while a catalyst-coated carbon paper served as the working electrode.

The working electrode was prepared as follows. The catalyst powder (1 mg) was dispersed in ethanol (400 μL) containing Nafion solution (5 wt%, 40 μL) by ultrasonication for 30 min to form a homogeneous ink. An aliquot (44 μL) of the ink was drop-cast onto carbon paper (geometric area: 1 cm × 1 cm), affording a catalyst loading of 0.1 mg cm^−2^, followed by drying at 30 °C. All potentials were converted to the reversible hydrogen electrode (RHE) scale using the equation:

1 $${\text{E }}\left( {{\mathrm{vs}}.{\text{ RHE}}} \right){ } = {\text{ E }}\left( {{\text{vs Hg}}/{\mathrm{HgO}}} \right){ } + { }0.0591{\text{ pH }} + { }0.098$$

Electrochemical measurements were taken in an Ar-saturated electrolyte containing 1 M KOH and 0.1 M KNO_3_ unless otherwise specified. Linear sweep voltammetry (LSV) was performed at a scan rate of 10 mV s^−1^ within a potential range from 0.1 to −1.0 V_RHE_, without iR correction. Electrochemical impedance spectroscopy (EIS) and Bode plots were measured over a frequency range of 0.01 Hz to 100 kHz with an amplitude of 10 mV at different potentials. Faradaic efficiencies (FE) and yields of NH_3_ were quantified from chronoamperometry (*i*–*t*) measurements at different applied potentials for 1h.

For membrane–electrode assembly (MEA) tests, a commercial two-electrode flow cell (Gaoss Union Photoelectric Technology Co.) was employed. The cathode was prepared by spray coating the catalyst onto porous hydrophilic carbon paper (TORAY TGP-H-060) with a geometric area of 2 cm × 2 cm. Specifically, 4 mg of catalysts was dispersed in ethanol (400 μL) and Nafion solution (5 wt%, 40 μL), followed by ultrasonication for 30 min to form a uniform ink. The obtained ink was all spray-coated onto carbon paper with a geometric area of 2 cm × 2 cm. The total catalyst loading on the cathode was 4 mg, corresponding to an area loading of 1 mg cm^−2^, while NiCoS_*x*_/NF was employed as the anode. All electrodes and the Nafion 211 proton exchange membrane were assembled with PTFE gaskets. The Nafion 211 effectively suppresses NO_3_^−^/NO_2_^−^ crossover, while the amount of NH_3_ crossing the membrane is negligible and does not affect the calculation of NH_3_ production. During operation, 1.0 M KOH was fed to the anodic compartment, while the cathodic compartment was supplied with 1.0 M KOH and 1.0 M KNO_3_. For the long-term MEA stability test, NiCo@NC was operated at a total current of − 1.5 A, corresponding to an actual current density of approximately − 0.4 A cm^−2^ based on the cathode geometric area of 4 cm^2^. During electrolysis, 500 mL of catholyte containing 1 M KOH and 1 M KNO_3_ was circulated through the cathodic compartment at a flow rate of 50 mL min^−1^, while 1 M KOH was used as the anolyte. To minimize nitrate depletion during continuous operation, the electrolyte was refreshed every 1h. All the MEA measurements were taken without iR compensation.

For both H-type cell and MEA measurements, the NH_3_ yield rate was normalized to the catalysts mass loaded on the working electrode:

2 $${\mathrm{r}}_{{{\mathrm{NH}}_{3} }} { } = \frac{{{\mathrm{n}}_{{{\mathrm{NH}}_{3} }} }}{{{\mathrm{t}} \times {\mathrm{m}}_{{{\mathrm{cat}}}} }}$$

where ${\mathrm{r}}_{{\mathrm{N}\mathrm{H}}_{3}}$ is the mass-normalized NH_3_ yield rate, ${\mathrm{n}}_{{\mathrm{N}\mathrm{H}}_{3}}$ is the amount of produced NH_3_, $\mathrm{t}$ is the electrolysis time, and ${\mathrm{m}}_{\mathrm{c}\mathrm{a}\mathrm{t}}$ is the catalyst mass loaded on the electrode.

### Coupled NO_3_^−^RR||MORR

A two-electrode flow cell electrolyzer was employed for coupled NO_3_^−^RR and methanol oxidation reaction (MORR). NiCo@NC deposited on porous hydrophilic carbon paper (1 cm × 1 cm) was used as the cathode, while NiCoS_*x*_/NF [40] coated on nickel foam (1 cm × 1 cm) served as the anode. The cathodic and anodic compartments were separated by a Nafion 211 proton exchange membrane. The catholyte consisted of 1 M KOH with 1 M KNO_3_, while the anolyte was 1 M KOH with 1 M methanol. All electrochemical measurements for the coupled NO_3_^−^RR||MORR system were taken without iR compensation.

The area-normalized formate yield rate was calculated as:

3 $${\mathrm{r}}_{{{\mathrm{HCOOH}}}} { } = \frac{{{\mathrm{n}}_{{{\mathrm{HCOOH}}}} }}{{{\mathrm{t}} \times {\mathrm{A}}}}$$

where ${\mathrm{r}}_{\mathrm{H}\mathrm{C}\mathrm{O}\mathrm{O}\mathrm{H}}$ is the area-normalized formate yield rate, ${\mathrm{n}}_{{\mathrm{N}\mathrm{H}}_{3}}$ is the amount of produced NH_3_, $\mathrm{t}$ is the electrolysis time, and $\mathrm{A}$ is the geometric electrode area.

The electrical energy input of different electrolysis systems was calculated as:

4 $${\mathrm{En}} = {\mathrm{U}}_{{{\mathrm{cell}}}} \times {\mathrm{I}} \times {\mathrm{t}}$$

where $\mathrm{E}\mathrm{n}$ is the area-normalized electrical energy input, ${\mathrm{U}}_{\mathrm{c}\mathrm{e}\mathrm{l}\mathrm{l}}$ is the cell voltage, $\mathrm{I}$ is the operating current, and t is the electrolysis time.

## Results and Discussion

### Construction and Structural Features of Carbon-Encapsulated NiCo Alloy Catalysts

**Fig. 1:**
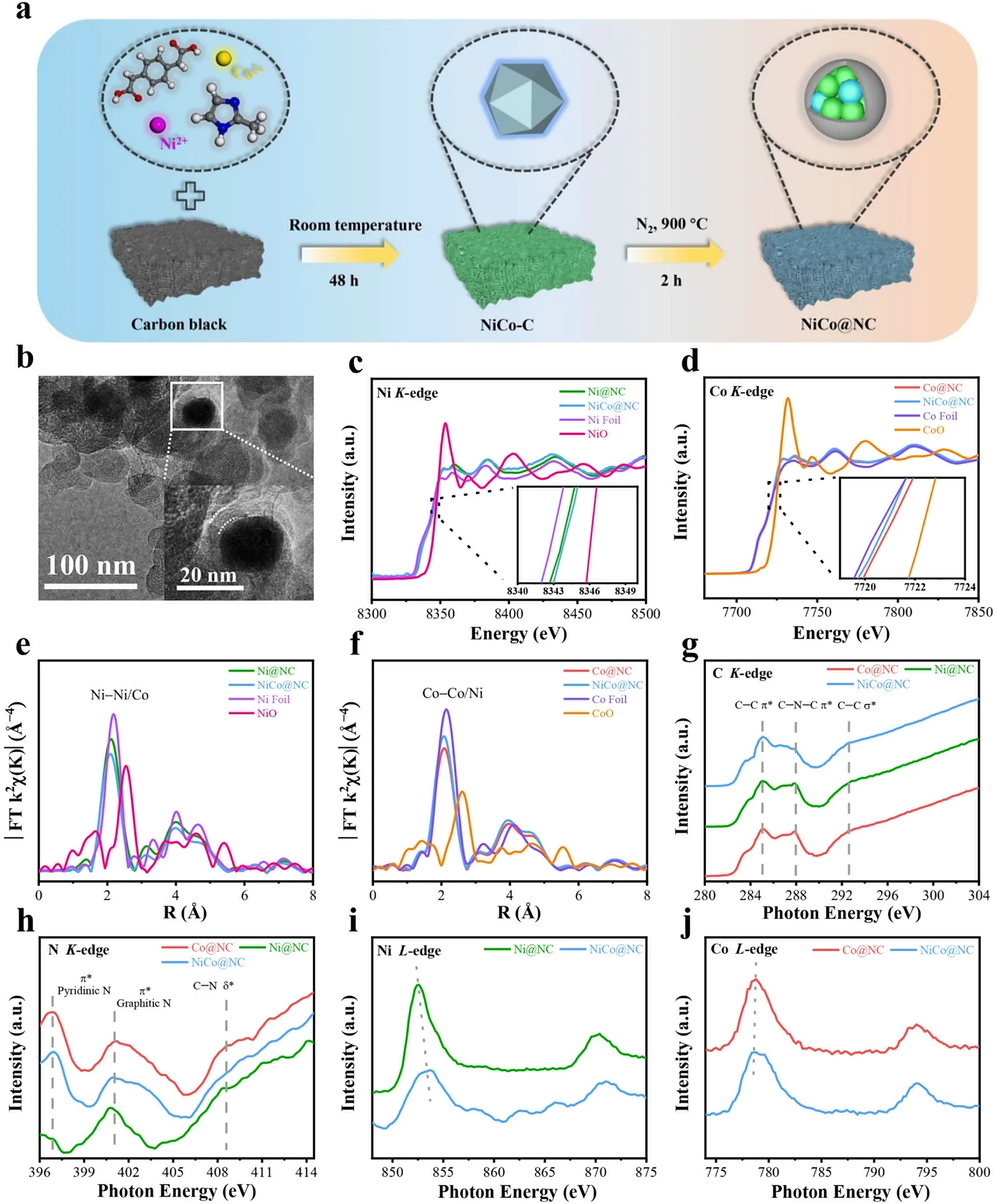
Structural characterization and electronic state analysis of Ni@NC, Co@NC, and NiCo@NC. **a** Schematic illustration of the synthesis of NiCo@NC. **b** TEM image of NiCo@NC. **c, d** Ni and Co *K*-edge XANES spectra. **e, f** *k*^3^-weighted FT-EXAFS spectra at the Ni and Co *K*-edges. **g, h** C and N *K*-edge XANES spectra of Co@NC, Ni@NC, and NiCo@NC. **i** Ni *L*-edge XANES spectra of Ni@NC and NiCo@NC. **j** Co *L*-edge XANES spectra of Co@NC and NiCo@NC

Figure 1a schematically illustrates the synthesis of the carbon-encapsulated NiCo alloy catalyst (denoted as NiCo@NC), which involves activation of commercial carbon black, deposition of a bimetallic precursor (NiCo-ZB), and subsequent high-temperature pyrolysis. For comparison, monometallic counterparts (Ni@NC and Co@NC) were synthesized under identical conditions. The powder X-ray diffraction (PXRD) patterns of the Co-ZB and NiCo-ZB precursors exhibit well-defined diffraction peaks characteristic of a crystalline MOF framework. The peaks observed at 10°, 18°, and 26° correspond to the (200), (222), and (510) planes in NiCo-ZB and Co-ZB, which are consistent with the characteristic planes of ZIF-67 [41]. This suggests that the Co-based precursor retains the structural features of ZIF-67. Additionally, the peaks at 12° and 24° correspond to the (010) and (020) planes in Ni-ZB, which are characteristic of Ni-based BDC framework (Ni-BDC) [42]. These results confirm the formation of NiCo-ZB as a bimetallic MOF precursor (Fig. S1). After pyrolysis, the PXRD patterns of NiCo@NC, Co@NC, and Ni@NC display a broad diffraction feature assigned to the (002) plane of graphitic carbon (Fig. S2). The diffraction peaks of Ni@NC can be indexed to the (111), (200), and (220) planes of face-centered cubic (fcc) metallic Ni (PDF/JCPDS No. 04-0850), while those of Co@NC also correspond to fcc metallic Co (PDF/JCPDS No. 15-0806). In addition, distinct diffraction peaks located at 44.2°, 51.5°, and 75.9° are observed for NiCo@NC. These peaks, originating from metallic NPs, appear at intermediate positions between those of monometallic Ni and Co, indicating the formation of alloyed NiCo NPs (Fig. S3) [43]. The formation of NiCo alloy is likely attributed to the homogeneous mixing of Ni and Co species in the MOF precursor, which facilitates their reduction and atomic interdiffusion during pyrolysis, while the organic ligands are carbonized into an N-doped carbon shell.

The porous structures of the catalysts were investigated by N_2_ sorption measurements at 77 K (Fig. S4). All samples exhibit combined type I and type IV isotherms, indicating the coexistence of micropores and mesopores. The corresponding pore size distribution curves (Fig. S5) show dominant pores below 2 nm, confirming the microporous nature of the materials. The surface areas range from 152 to 186 m^2^ g^−1^, with total pore volumes of 0.34–0.48 cm^3^ g^−1^ (Table S1). Scanning electron microscopy (SEM) images reveal irregularly aggregated porous particles (Fig. S6). Transmission electron microscopy (TEM) images further reveal that NiCo alloy NPs are encapsulated within carbon layers (Fig. 1b) with metal NPs’ average particle sizes of 30.4, 19.7, and 28.4 nm for NiCo@NC, Ni@NC, and Co@NC, respectively (Fig. S7). Notably, a conformal graphitic carbon shell with an interlayer spacing of approximately 0.37 nm, assignable to the (002) plane, is clearly observed surrounding the metallic cores. Similar core–shell features are also present in Ni@NC and Co@NC, indicating that the carbon shell originates from the carbonization of organic ligands in the MOF precursors [44]. High-resolution TEM shows a lattice spacing of 0.208 nm for NiCo@NC, corresponding to the NiCo (111) plane, which is intermediate between those of Ni (111) (0.204 nm) and Co (111) (0.209 nm), further confirming the formation of the NiCo alloy (Fig. S8) [45]. Elemental mapping analysis demonstrates a homogeneous spatial distribution of Ni and Co throughout NiCo@NC (Fig. S9). Inductively coupled plasma optical emission spectrometry (ICP–OES) analysis (Table S2) shows Ni contents of 2.7 and 27.1 wt% for NiCo@NC and Ni@NC, respectively, while the Co contents are 28.9 and 26.3 wt% for NiCo@NC and Co@NC, respectively. Elemental analysis shows that NiCo@NC contains 59.9 wt% C and 0.4 wt% N, with a calculated N–Ni–Co molar ratio of 1:1.61:17.17 (Table S3).

Raman spectra (Fig. S10) exhibit two characteristic peaks at 1341 and 1592 cm^−1^, corresponding to the D and G bands for the carbon shell [30]. The intensity ratio (*I*_D_/*I*_G_) is 1.09 for NiCo@NC, slightly higher than those of Ni@NC (1.02) and Co@NC (1.01), suggesting a higher density of structural defects in NiCo@NC. X-ray photoelectron spectroscopy (XPS) survey spectra of Co@NC, Ni@NC, and NiCo@NC (Fig. S11) reveal the presence of C 1*s*, N 1*s*, and O 1*s* signals for all samples, while Ni 2*p* and Co 2*p* signals are detected for Ni@NC and Co@NC, respectively. NiCo@NC predominantly exhibits Co 2*p* signals, with no discernible Ni 2*p* peak, which can be attributed to the relatively low surface exposure of Ni species. High-resolution C 1*s* spectra (Fig. S12) can be deconvoluted into three components at 284.7, 285.2, and 286.4 eV, corresponding to *sp*^2^ C, *sp*^3^ C, and C–N bonds [46], respectively, confirming the graphitic nature of the carbon shell. The N 1s spectra (Fig. S13) reveal the coexistence of pyridinic-N and graphitic-N species. In the Ni 2*p*_3/2_ region (Fig. S14), Ni@NC displays peaks at 852.5 and 854.5 eV, attributable to metallic Ni^0^ and oxidized Ni^2+^ species [47]. Meanwhile, Co@NC and NiCo@NC show Co 2*p*_3/2_ peaks at 778.1 eV (Co^0^) and 780.4 eV (Co^2+^) (Fig. S15), indicating the coexistence of metallic and oxidized Co species [48].

X-ray absorption fine structure (XAFS) spectra were collected. The Ni *K*-edge X-ray absorption near-edge structure (XANES) spectra (Fig. 1c) show that the absorption edges of Ni in NiCo@NC and Ni@NC are close to that of Ni foil, indicating predominantly metallic Ni species. A similar trend is observed in the Co *K*-edge XANES spectra (Fig. 1d), indicating the domination of metallic Co species. Importantly, the rising-edge positions follow the sequence Ni foil < Ni@NC < NiCo@NC << NiO for the Ni *K*-edge and Co foil < NiCo@NC < Co@NC << CoO for the Co *K*-edge, suggesting charge redistribution and electron transfer from Ni to Co upon alloy formation. Fourier transform extended X-ray absorption fine structure (EXAFS) spectra at the Ni *K*-edge (Fig. 1e) and Co *K*-edge (Fig. 1f) show the peaks at approximately 2.0 Å, corresponding to Ni–Ni/Co and Co–Co/Ni scattering paths, respectively. EXAFS fitting (Figs. S16 and S17, Tables S4 and S5) yields coordination numbers of 10.9 and 10.1 for the Co–Co/Ni shell in NiCo@NC and Co@NC, 11.2 and 11.8 for the Ni–Ni/Co shell in NiCo@NC and Ni@NC, respectively, with comparable bond lengths of ~2.48 Å, confirming the formation of a well-alloyed NiCo structure.

Soft X-ray absorption spectroscopy (XAS) further elucidates the electronic interaction between the alloy core and the carbon shell. The C *K*-edge XANES spectra (Fig. 1g) display characteristic peaks at 285.5 and 288.2 eV, corresponding to π* C–C transitions in graphitic carbon and π* C–N–C antibonding orbitals, respectively [49, 50]. The N *K*-edge spectra (Fig. 1h) exhibit features assignable to pyridinic-N (~397 eV), graphitic-N (~401 eV), and C–N (~408.5 eV) [51]. Furthermore, the Ni *L*-edge XANES spectra (Fig. 1i) reveal an increased Ni valence state upon alloying with Co, while the Co *L*-edge spectra (Fig. 1j) show a slightly reduced Co valence state in NiCo@NC relative to Co@NC, confirming strong electronic coupling and charge redistribution within the NiCo alloy encapsulated by the N-doped carbon shell.

### Selective Electrocatalytic NO_3_^−^RR Enabled by NiCo@NC

**Fig. 2:**
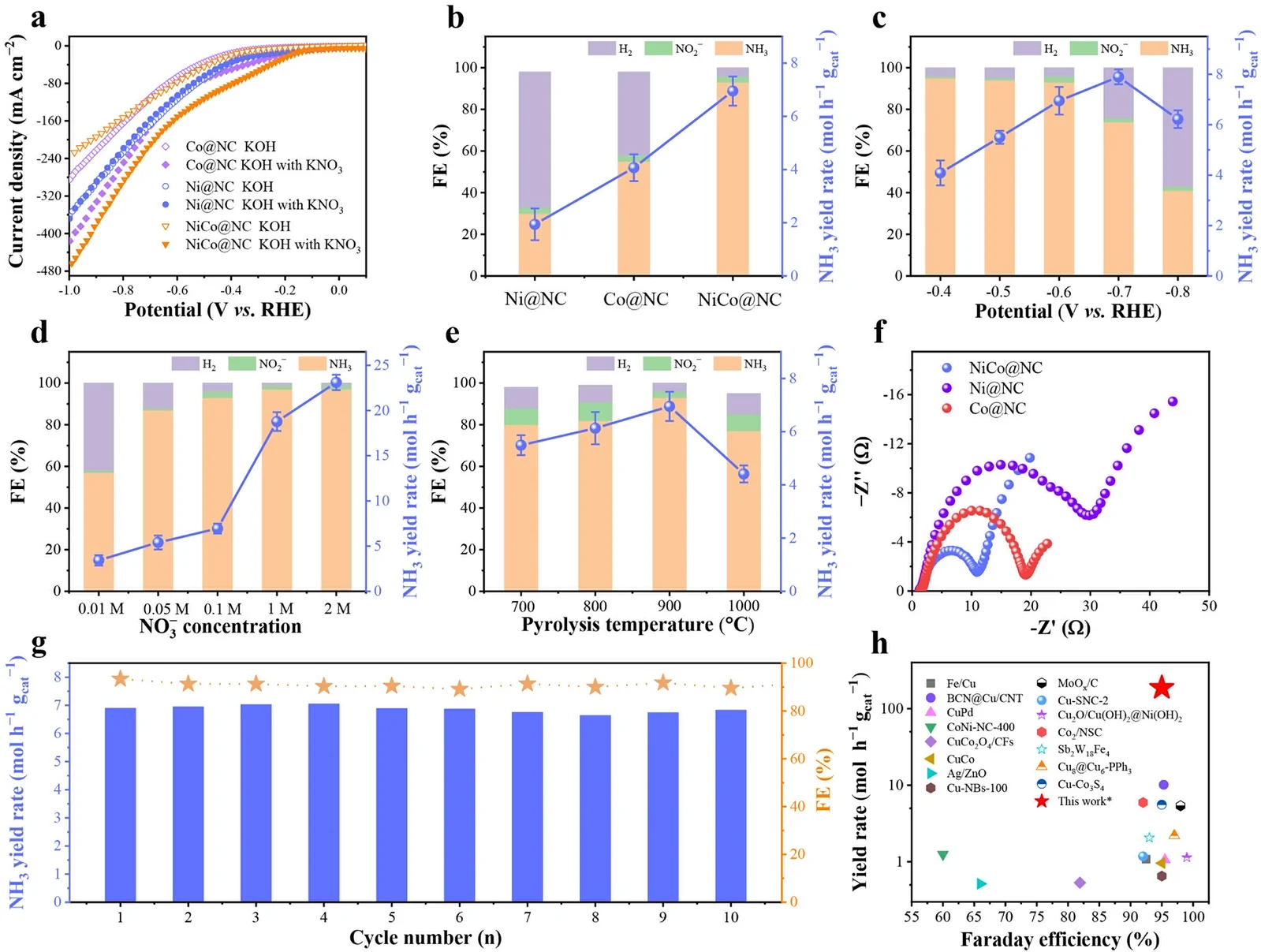
Electrochemical NO_3_^−^RR performance of NiCo@NC, Co@NC, and Ni@NC in an H-type cell. **a** LSV curves recorded in 1 M KOH with and without 0.1 M KNO_3_. **b** NH_3_ FE and yield rate over different catalysts. Influence of **c** applied potential, **d** nitrate concentration, and **e** pyrolysis temperature on the NO_3_^−^RR performance of NiCo@NC. **f** Nyquist plots of different catalysts. **g** Cycling stability test of NiCo@NC for NO_3_^−^RR. Unless otherwise specified, the reaction conditions were: catalyst loading, 0.1 mg; electrolysis time, 1 h; electrolyte, 1 M KOH containing 0.1 M KNO_3_; applied potential, −0.6 V_RHE_. **h** Benchmark comparison of NH_3_ FE and mass-normalized NH_3_ yield rate. NiCo@NC was tested in 1 M KOH with 1 M KNO_3_ at −0.6 V_RHE_; The reported literature data were collected under 1 M KOH with 0.1 M KNO_3_ or 0.5 M K_2_SO_4_, with 0.1 M KNO_3_, and the corresponding applied potentials are summarized in Table S10

The electrocatalytic NO_3_^−^RR performance was first evaluated in an H-type cell and the corresponding calibration curves for the target products and by-products are presented in Fig. S18 and Table S6. Linear sweep voltammetry (LSV) curves of NiCo@NC, Ni@NC, and Co@NC were recorded in 1.0 M KOH electrolyte in the absence and presence of 0.1 M KNO_3_ (Fig. 2a). In nitrate-free electrolyte, the cathode current density increases continuously with increasingly negative potentials, following the order Ni@NC > Co@NC > NiCo@NC, reflecting their relative HER activities. Upon the introduction of nitrate, all catalysts exhibit markedly enhanced current densities, with the activity order reversed to Ni@NC < Co@NC < NiCo@NC. This contrast clearly demonstrates a markedly enhanced preference of NiCo@NC toward NO_3_^−^RR over HER under identical conditions. The product distribution and selectivity of the catalysts are summarized in Figs. 2b and S19a. NiCo@NC achieves a high NH_3_ FE of 93%, significantly exceeding those of Co@NC (55%) and Ni@NC (30%), while exhibiting minimal FE for H_2_ (4%) and NO_2_^−^ (3%). Correspondingly, the NH_3_ yield rate of NiCo@NC reaches 6.95 mol h^−1^ g_cat_^−1^, outperforming Co@NC (4.07 mol h^−1^ g_cat_^−1^) and Ni@NC (1.94 mol h^−1^ g_cat_^−1^). To clarify the role of the conductive carbon support, a carbon black-free analogue (NiCo@NC-NB) was prepared for comparison (Fig. S20). Although NiCo@NC-NB delivers a comparable NH_3_ FE of 90%, its NH_3_ yield rate decreases to 4.35 mol h^−1^ g_cat_^−1^ (Fig. S21), highlighting the essential role of conductive carbon black in enabling higher attainable current densities.

The potential dependence of NO_3_^−^RR over NiCo@NC was further investigated (Figs. 2c and S19b). A volcano-shaped relationship between NH_3_ yield rate and applied potential is observed, with optimal performance achieved at − 0.6 V_RHE_. The influence of nitrate concentration was examined by varying the KNO_3_ concentration in the electrolyte (Fig. S22). Increasing nitrate concentration leads to continuous enhancement in current density, NH_3_ FE, and yield rate, reaching a maximum NH_3_ yield rate of 23.1 mol h^−1^ g_cat_^−1^ with an FE of 97% in 2.0 M KNO_3_ electrolyte (Figs. 2d and S19c). Under the optimized conditions, NiCo@NC achieved the highest NH_3_ yield rate of 187 mol h^−1^ g_cat_^−1^ with an FE of 95% at an estimated current density of ~420 mA cm^−2^ during chronoamperometric electrolysis at − 0.6 V_RHE_ for 1h, using 1 M KOH containing 1 M KNO_3_ as the electrolyte and a catalyst loading of 0.01 mg on carbon paper. To further evaluate the wastewater remediation potential of NiCo@NC, NO_3_^−^RR tests were conducted in a KOH-free, carbonate-containing electrolyte composed of 0.5 M Na_2_SO_4_, 0.1 M Na_2_CO_3_, and 0.01 to 0.1 M NaNO_3_. As shown in Fig. S23, NiCo@NC delivers an NH_3_ FE of 83% and an NH_3_ yield rate of 1.29 mol h^−1^ g_cat_^−1^ at 0.1 M NaNO_3_, and still maintains an NH_3_ FE of 62% at 0.01 M NaNO_3_, demonstrating its nitrate reduction capability under diluted carbonate-containing conditions. The effect of pyrolysis temperature on catalytic performance was also evaluated (Figs. 2e and S19d). Both NH_3_ yield and FE increase as the annealing temperature rises from 700 to 900 °C, but decrease sharply at 1000 °C, which can be attributed to excessive aggregation of NiCo NPs and partial collapse of the graphitic carbon structure at over elevated temperatures. Accordingly, 900 °C was identified as the optimal pyrolysis temperature. Isotopic labeling experiments using ^15^NO_3_^−^ confirm that the produced NH_3_ originates exclusively from nitrate electroreduction, as evidenced by the characteristic doublet signals of ^15^NH_4_^+^ and triplet signals of ^14^NH_4_^+^ in the ^1^H NMR spectra (Fig. S24) [52, 53]. Nitrogen balance analysis after 1 h of NO_3_^−^RR shows a NO_3_^−^ conversion of 15.9%, with NH_3_ and NO_2_^−^ yields of 13.9% and 1.8%, respectively, while no detectable N_2_ by-product is observed, demonstrating a well-balanced nitrogen distribution (Fig. S25).

Electrochemical impedance spectroscopy (EIS) analysis (Fig. 2f) reveals that NiCo@NC exhibits the smallest Nyquist semicircle, indicating the fastest interfacial charge transfer kinetics. The Tafel slope of NiCo@NC (190 mV dec^−1^) is significantly lower than those of Co@NC (287 mV dec^−1^) and Ni@NC (790 mV dec^−1^) (Fig. S26), reflecting more favorable apparent reaction kinetics for NO_3_^−^RR. In addition, NiCo@NC displays the largest double-layer capacitance (C_dl_), corresponding to the highest electrochemically active surface area (ECSA) (Fig. S27). Long-term cycling tests demonstrate stable NH_3_ production over NiCo@NC at − 0.6 V_RHE_ (Fig. 2g), confirming its excellent durability. The structural stability of NiCo@NC after long-term electrolysis was confirmed by SEM, TEM, and XRD characterizations. As shown in Fig. S6g, h, the spent NiCo@NC retains its aggregated porous morphology, and the NiCo NPs remain well dispersed with an average diameter of 31.3 nm, showing no obvious particle growth compared with the fresh catalyst (Fig. S28). In addition, the XRD pattern of spent NiCo@NC remains consistent with that of the fresh catalyst, with the characteristic peaks of graphitic carbon and metallic NiCo alloy preserved (Fig. S29). These results confirm the good structural stability of NiCo@NC during NO_3_^−^RR. In contrast, both the NH_3_ yield rate and FE of Co@NC decline markedly after 5 cycles (Fig. S30), indicating that Ni incorporation plays a critical role in enhancing stability in NiCo@NC. A comprehensive benchmarking comparison (Fig. 2h) shows that NiCo@NC outperforms previously reported NO_3_^−^RR catalysts by achieving a uniquely favorable combination of ultrahigh NH_3_ yield rate and high FE.

### High-Current-Density Nitrate Reduction in MEA Configurations

**Fig. 3:**
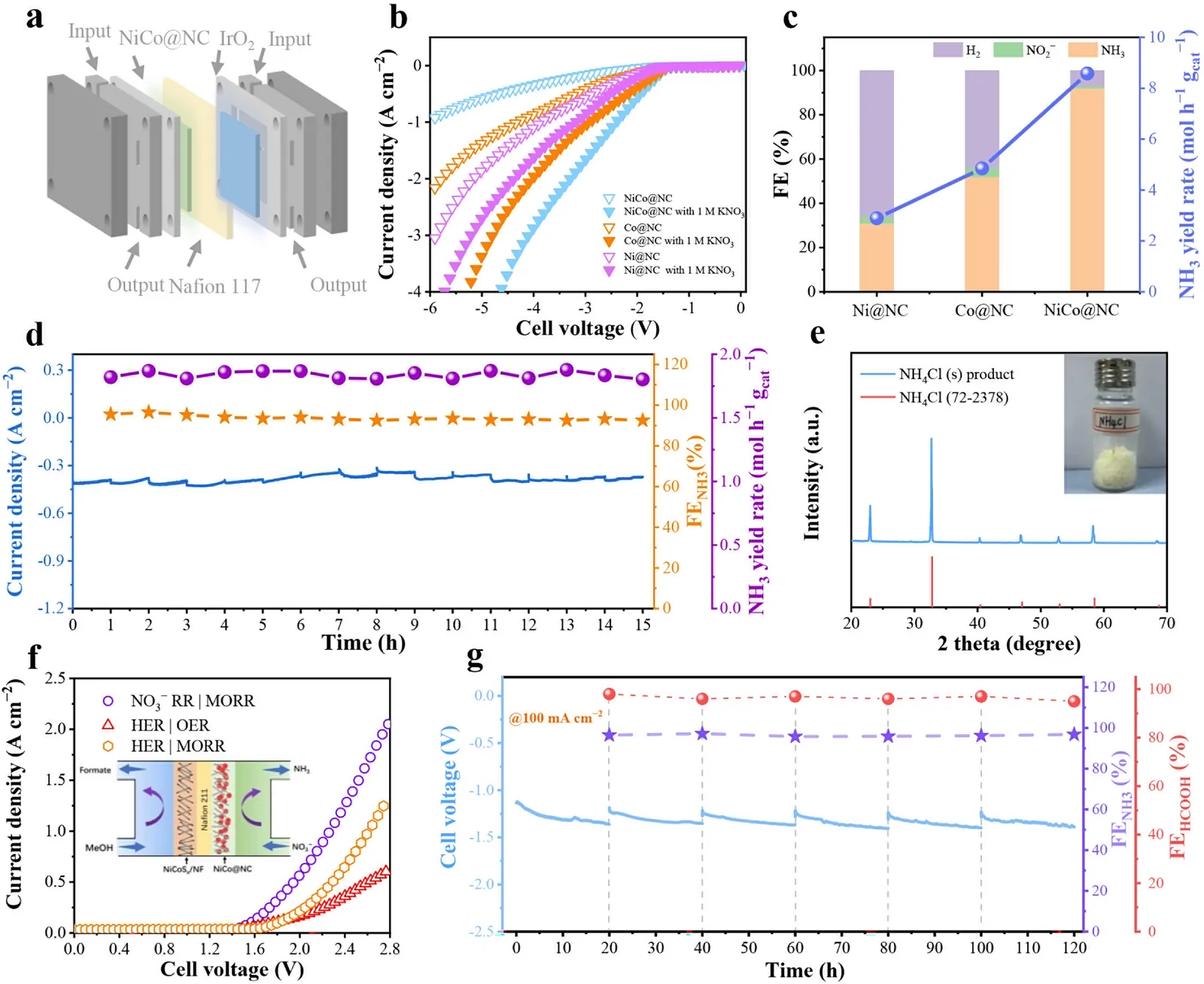
High-current NO_3_^−^RR performance in a MEA and paired electrolysis system. **a** Schematic illustration of the MEA flow reactor for NO_3_^−^RR. **b** LSV curves of NiCo@NC, Co@NC, and Ni@NC in 1 M KOH with 1 M KNO_3_ (solid lines) and without KNO_3_ (dotted lines). **c** NH_3_ FE and yield rate over different catalysts in the MEA reactor. **d** Chronoamperometric response, NH_3_ FE, and yield rate of NiCo@NC during repeated electrolysis at 1.8 V. **e** XRD pattern of the collected NH_4_Cl product. **f** LSV curves of the NO_3_^−^RR‖MORR, HER‖MORR, and HER‖OER systems in the MEA flow reactor. **g** Chronoamperometric curves together with the NH_3_ and HCOOH FEs during coupled cathodic NO_3_^−^RR on NiCo@NC and anodic MOR on NiCoS_x_/NF at 100 mA cm^−2^ with 1 cm^2^ electrode area. Unless otherwise specified, the reaction conditions were: catalyst loading, 1 mg cm^−2^; electrolysis time, 0.25 h; current density, 2.0 A cm^−2^; electrode area, 4 cm^2^; catholyte, 1 M KOH containing 1 M KNO_3_

To examine the scalability and industrial relevance of NiCo@NC, nitrate reduction was further investigated in a MEA configuration under high current density (Fig. 3a). LSV curves of NiCo@NC, Ni@NC, and Co@NC show a pronounced increase in current density upon the addition of 1.0 M KNO_3_ (Fig. 3b), following the same activity trend observed in H-type cell measurements. Among the tested catalysts, NiCo@NC exhibits the highest current response. At a current intensity of 2.0 A cm^−2^, NiCo@NC delivers an NH_3_ yield rate of 8.58 mmol h^−1^ g_cat_^−1^ with a high FE of 92% (Fig. 3c), significantly outperforming Ni@NC and Co@NC. Compared with recently reported ampere-level NO_3_⁻RR catalysts [54–56], which are predominantly based on exposed metal or metal oxide active surfaces, NiCo@NC extends high-current nitrate reduction to a carbon-encapsulated alloy interface while retaining high NH_3_ selectivity in MEA operation, highlighting the structural and operational advantages of the alloy@carbon architecture. Continuous electrolysis at – 400 mA cm^−2^ maintains stable NH_3_ production over 15h without noticeable decay (Fig. 3d), demonstrating the robustness and durability of NiCo@NC under sustained high-current operation.

The electrochemically generated NH_3_ was further collected using an acid-trapping method (Fig. S31). Subsequent evaporation of the trapping solution yields high-purity NH_4_Cl as a crystalline solid (Fig. 3e), confirming efficient NH_3_ recovery and validating the practical feasibility of the process. Furthermore, cathodic NO_3_^−^RR over NiCo@NC was coupled with anodic methanol oxidation to formate (MORR), catalyzed by the previously reported NiCoSₓ/NF catalyst [40], in a two-electrode flow cell electrolyzer. As shown in Fig. 3f, the NO_3_^−^RR||MORR system reduces the overall cell voltage by approximately 0.7 V compared with the conventional OER||HER configuration and by approximately 0.4 V compared with the HER||MORR system, demonstrating its improved energy efficiency and superior paired electrolysis performance. Meanwhile, the area-normalized energy inputs of the NO_3_^−^RR||MORR, HER||MORR, and OER||HER systems are 1.20, 1.44, and 1.62 Wh cm^−2^ at 0.6 A cm^−2^, respectively, demonstrating the energy-saving advantage of the paired NO_3_^−^RR||MORR configuration. Under a high current density of 2.1 A cm^−2^, the coupled system achieves a cathode NH_3_ FE of 90% with a productivity of 8.8 mol h^−^^1^ g_cat_^−1^, along with an anodic formate FE of 96% and a productivity of 18.8 mmol h^−^^1^ cm^−2^ (Fig. S32). Compared with recently reported MEA-based NO_3_^−^RR catalysts, NiCo@NC exhibits competitive high-current-density operation and high NH_3_ FE, highlighting its advantage for membrane–electrode nitrate-to-ammonia conversion (Fig. S33 and Table S7). Notably, the NO_3_^−^RR‖MORR configuration maintains a stable cell voltage over prolonged operation exceeding 120h at 100 mA cm^−2^ (Fig. 3g), highlighting its excellent operational stability and strong potential for integrated ammonia and value-added chemical co-production.

### *In Situ* Spectroscopic Identification of Reaction Intermediates and Active Site Stability

**Fig. 4:**
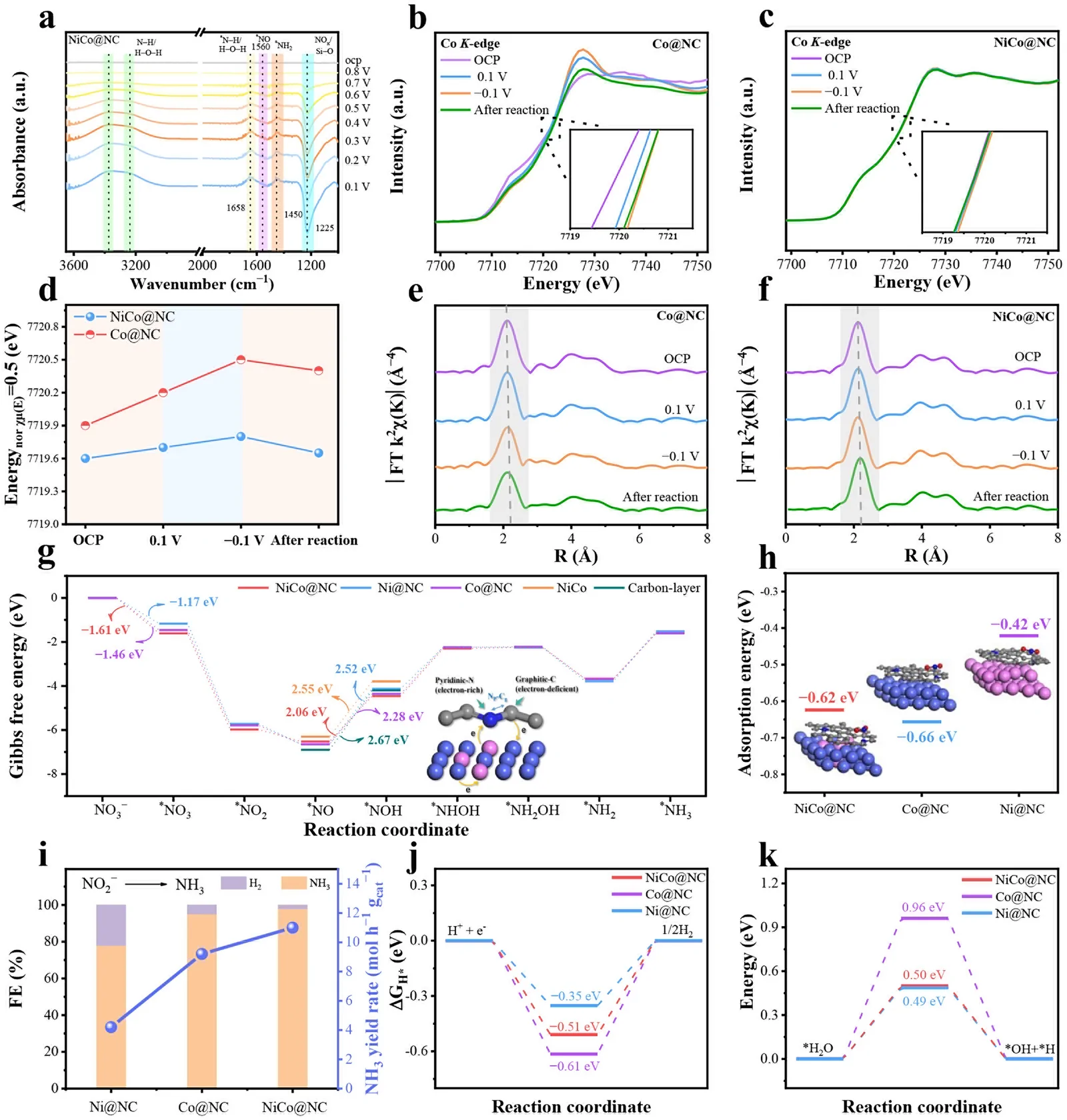
*Operando* spectroscopic and theoretical analysis of the NO_3_^−^RR mechanism over NiCo@NC.** a** *In situ* FT-IR spectra of NiCo@NC during NO_3_^−^RR. **b, c** *Operando* Co *K*-edge XANES spectra of Co@NC and NiCo@NC in 1 M KOH containing 0.1 M KNO_3_. **d** Normalized absorption edge energy [χμ(E) = 0.5] of Co@NC and NiCo@NC as a function of applied potential. **e, f** *Operando* Co *K*-edge FT-EXAFS spectra of Co@NC and NiCo@NC in 1 M KOH containing 0.1 M KNO_3_. **g** Gibbs free energy profiles for NO_3_^−^RR over NiCo@NC, NiCo, carbon layer, Ni@NC, and Co@NC. The inset shows the N_p_–C_g_ configuration used to model the active sites in NiCo@NC. **h** Adsorption energies of nitrite on NiCo@NC, Ni@NC, and Co@NC. **i** NH_3_ FE and yield rate over different catalysts for NO_2_^−^RR. Reaction conditions: catalyst loading, 0.1 mg; applied potential, −0.6 V_RHE_; electrolysis time, 1 h; electrolyte, 1 M KOH containing 0.1 M KNO_2_. **j** Gibbs free energy profiles for HER over NiCo@NC, Co@NC, and Ni@NC. **k** Gibbs free energy profiles for water dissociation over NiCo@NC, Co@NC, and Ni@NC

*In situ* Fourier transform infrared (*in situ* FT-IR) spectra recorded during NO_3_^−^RR over NiCo@NC, Ni@NC, and Co@NC (Figs. 4a, S34, and S35) reveal the evolution of surface-bound intermediates as the applied potential varies. For all catalysts, characteristic vibrational bands related to hydrogenated nitrogen species are observed at approximately 1450 and 1658 cm^−1^, which can be assigned to adsorbed *NH_2_/*NH_x_ species, while possible overlap with interfacial water bending vibrations is also considered [57, 58]. With increasing applied potential, a broad band centered at ~3400 cm^−1^ gradually emerges and intensifies, corresponding to N–H/H–O–H stretching vibrations and indicating the progressive formation of hydrogenated products. In addition, a distinct band at ~1560 cm^−1^, associated with surface-bound *NO/NO_x_ intermediates [59–61], appears prominently on NiCo@NC but is much weaker or absent on Ni@NC and Co@NC, suggesting preferential accumulation and stabilization of *NO-related intermediates on NiCo@NC during NO_3_^−^RR. The absence of a clear *NOH signal can be attributed to its short lifetime, low surface coverage, and possible peak overlap; once formed after the rate-determining *NO → *NOH step, *NOH is rapidly hydrogenated to *NH_2_ and *NH_3_, making direct FT-IR detection difficult. Moreover, NiCo@NC exhibits markedly stronger NO_3_^−^-related and *NH_3_-related signals at higher potentials compared with Ni@NC and Co@NC, mirroring its higher NO_3_^−^RR activity. Online differential electrochemical mass spectrometry (DEMS) was further performed to verify the reaction pathway during NO_3_^−^RR over NiCo@NC. As shown in Fig. S36, potential-responsive signals corresponding to NO, NOH, NH, NH_2_, and NH_3_ are observed, whereas no obvious signals of N–N coupling products such as N_2_ and N_2_O are detected. These results confirm that the NO_3_^−^RR pathway over NiCo@NC is mainly directed toward stepwise hydrogenation to NH_3_ rather than N–N coupling.

To probe catalyst stability under operating conditions, *operando* XAFS measurements were further taken to track the electronic state evolution of Co species in NiCo@NC and Co@NC during NO_3_^−^RR (Fig. 4b–f). As increasingly negative potentials are applied, the Co *K*-edge XANES spectra of Co@NC display a gradual positive shift of the absorption edge relative to the open-circuit potential (OCP), indicating a progressive increase in the Co oxidation state (Fig. 4b). In sharp contrast, NiCo@NC shows negligible edge shifts across the same potential range, indicating a markedly different potential-dependent valence evolution of Co species in NiCo@NC compared with Co@NC (Fig. 4c). Comparison of the edge position evolution further shows that the Co valence in Co@NC increases during electrolysis and does not fully recover after the reaction, whereas the one in NiCo@NC shows minor variation under different potentials and recovered to initial state after electrolysis (Fig. 4d). EXAFS fitting shows that the Co–Co bond length of Co@NC expands from 2.55 at OCP to 2.59 Å at – 0.1 V_RHE_ and remains elongated after electrolysis, indicative of irreversible structural distortion and reconstruction (Figs. 4e and S37, Table S8). By contrast, the Co–Co coordination environment in NiCo@NC remains nearly unchanged throughout the entire electrochemical process (Figs. 4f and S38, Table S9). Thus, the spectroscopic observations reveal that the alloy effect prevents the potential-induced Co oxidation and reconstruction, enhancing the stability of metal–carbon interface.

### Interfacial Electronic Regulation and Synergistic Mechanism for Efficient NO_3_^−^RR

Building on the above spectroscopic evidence, density functional theory (DFT) calculations were conducted to elucidate how interfacial electronic regulation at the NiCo alloy–carbon interface governs NO_3_^−^RR behavior. A periodic core–shell model comprising an fcc NiCo alloy core encapsulated by an N-doped graphitic carbon layer was constructed to represent the active structure of NiCo@NC, while analogous models with single-metal cores (Ni or Co) were employed as references for Ni@NC and Co@NC. Electronic structure analysis (Fig. S39) shows that the *d*-band centers of both Ni and Co in NiCo@NC shift closer to the Fermi level (E_f_) relative to those in Ni@NC and Co@NC, indicating increased electronic reactivity induced by alloying. Charge density difference maps and Bader charge analysis (Fig. S40) further reveal pronounced interfacial charge redistribution between the alloy core and carbon shell. Specifically, graphitic carbon domains exhibit electron-donating behavior, whereas pyridinic-N sites act as electron acceptors. Quantitatively, Ni atoms withdraw ~0.82 e^−^ from the carbon framework, while Co atoms promote electron accumulation (~0.70 e^−^) at neighboring pyridinic-N sites. As a consequence, the alloy–carbon interface produces spatially polarized N_p_–C_g_ domains (Fig. S41 and the inset image in Fig. 4g), featuring more electron-deficient graphitic-C and more electron-rich pyridinic-N regions, which provides a rational basis for dual-site catalytic behavior. To identify the representative adsorption site in NiCo@NC, NO_3_^−^, NO_2_^−^, and NO adsorption were compared at pyridinic-N, graphitic-C, and graphitic-N sites. Pyridinic-N shows the strongest adsorption for these nitrogen-containing species, owing to its electron-rich and defect-like local environment, and was therefore used for subsequent NO_3_^−^RR free energy calculations (Figs. S42–S44).

The Gibbs free energy profiles of NO_3_^−^RR (Fig. 4g) show that nitrate adsorption is exothermic on all catalysts, while NiCo@NC displays the strongest NO_3_^−^ adsorption (− 1.61 eV). Importantly, the calculated rate-determining step (*NO **→** *NOH; Figs. S45–S47) exhibits the lowest energy barrier on NiCo@NC (2.06 eV), compared with Ni@NC (2.52 eV) and Co@NC (2.28 eV), in agreement with the experimentally observed activity trend. Beyond nitrate adsorption, NiCo@NC and Co@NC both show stronger nitrite adsorption energies (− 0.62 and − 0.66 eV, respectively) than Ni@NC (− 0.42 eV) (Fig. 4h), indicating that Co-containing catalysts preferentially stabilize *NO_2_, facilitating its further conversion and suppressing nitrite desorption as a by-product [62]. This prediction is consistent with the experimentally observed activity sequence for nitrite reduction (NiCo@NC > Co@NC >> Ni@NC) at − 0.6 V_RHE_ in 1.0 M KOH containing 0.1 M KNO_2_ (Figs. S48 and 4i). In addition, NiCo@NC presents the most favorable *NO adsorption energy (− 0.53 eV) (Fig. S49), which rationalizes the *in situ* FT-IR observation of a distinct *NO band on NiCo@NC and supports the lowered barrier for the key *NO hydrogenation step. Hydrogen-involving elementary steps were further examined to clarify how NiCo@NC maintains high NH_3_ selectivity while suppressing HER (Fig. 4j, k). For HER, Ni@NC exhibits the weakest proton binding (− 0.35 eV), corresponding to facile H_2_ formation and thus pronounced HER activity. In contrast, Co@NC binds H* too strongly (− 0.61 eV), which suppresses HER but can also impede proton release and utilization for NO_3_^−^RR-relevant intermediates hydrogenation. NiCo@NC exhibits a moderate proton adsorption free energy (−0.51 eV), providing a balanced H* coverage that supports hydrogenation steps in NO_3_^−^RR while avoiding excessive H_2_ evolution. Water dissociation barriers over Ni@NC and NiCo@NC (0.49 and 0.50 eV, respectively) are substantially lower than that of Co@NC (0.96 eV) (Fig. 4k), indicating that Ni incorporation enhances H_2_O activation and proton generation.

To isolate the contribution of the alloy–carbon interface, additional calculations were performed on bare NiCo alloy NPs and stand-alone N-doped carbon layers. Both systems exhibit significantly higher barriers for the *NO **→** *NOH step (2.55 and 2.67 eV, respectively; Fig. 4g), demonstrating that neither the alloy core nor the carbon shell alone can account for the accelerated NO hydrogenation. Moreover, adsorption energies of NO_3_^−^, NO_2_^−^, and NO on bare NiCo or isolated carbon layers are weaker than those on NiCo@NC (Figs. S50–S52), indicating that the interfacial electronic coupling uniquely strengthens binding at the N_p_–C_g_ sites adjacent to metal atoms. Bare NiCo also shows a higher water dissociation barrier (0.58 eV) than NiCo@NC (Fig. S53), further evidencing that carbon encapsulation facilitates proton generation at the metal surface.

The combined spectroscopic and theoretical results converge to show that the outstanding performance of NiCo@NC arises from the synergistic integration of electronic regulation, interfacial stability, and efficient charge transport enabled by its alloy–carbon core–shell architecture. The Ni species in the NiCo alloy core plays a crucial electronic regulation role by donating electrons to Co, induces Ni → Co charge transfer, which enriches the Co centers and simultaneously stabilizes them against oxidation and reconstruction under cathodic polarization, as evidenced by the reversible Co valence and coordination observed in *operando* XAFS measurements. This Ni-driven electronic modulation not only promotes the formation of electron-enriched Co species, but also strengthens the electronic coupling between the NiCo alloy core and the N-doped graphitic shell, thereby enabling efficient electron transport, and more importantly, creates charge-polarized domains, electron-deficient graphitic carbon, and electron-rich pyridinic-N that spatially couple fast water dissociation/proton supply with strong adsorption and activation of nitrogen intermediates. This interfacial configuration rationalizes the *in situ* observed accumulation of *NO intermediates and lowers the energetic penalty of the rate-determining NO → NOH step, while maintaining a balanced H coverage. As a result, HER is effectively suppressed and high NH_3_ selectivity is sustained, particularly under high-current-density and MEA operating conditions.

## Conclusions

In summary, an N-doped carbon-encapsulated NiCo alloy catalyst (NiCo@NC) enables selective and durable nitrate-to-ammonia electrosynthesis at technologically relevant, ampere-level current densities. NiCo@NC exhibits high NH_3_ selectivity and productivity in H-type cells (187 mol h^−1^ g_cat_^−1^, 95% FE) and, more importantly, sustains ampere-level current densities of 2.0 A cm^−2^ with a high FE of 92% in MEA configurations. When integrated into a paired electrolyzer with anodic methanol oxidation, the system further enables simultaneous NH_3_ (90% FE) and formate (96% FE) production at reduced cell voltage and 2.1 A cm^−2^, highlighting its compatibility with energy-efficient co-electrolysis. Mechanistically, *operando* spectroscopic and theoretical analyses reveal that alloy–carbon interfacial regulation is central to this device-level performance. Ni → Co charge transfer stabilizes Co active sites against oxidation and reconstruction, while the N-doped carbon shell provides high electronic conductivity and generates charge-polarized domains that decouple proton supply from intermediate hydrogenation. This interfacial architecture enables fast water dissociation, controlled proton flux, and selective stabilization of key nitrogen intermediates, thereby suppressing HER and sustaining high NH_3_ selectivity even at large current densities. Together, these results establish alloy@NC interfacial engineering as a practical and scalable design strategy for high-current, multi-electron electrochemical systems, bridging fundamental interfacial regulation with device-level performance for pollutant-to-chemical conversion.

## Supplementary Information

Below is the link to the electronic supplementary material.

Supplementary file1 (DOCX 39114 KB)
