# Supplementary material for: Interfacial Electronic Regulation in N-Doped Carbon-Encapsulated NiCo Alloy Enables High-Current Nitrate-to-Ammonia Electrosynthesis
Source: Nanomicro Lett. 2026 Nov 9;19:58. doi: 10.1007/s40820-026-02359-6 (PMC13745612; doi:10.1007/s40820-026-02359-6)
Supplement: Supplementary file 1 [file 40820_2026_2359_MOESM1_ESM.docx]

Supporting Information for

**Interfacial Electronic Regulation in N-Doped Carbon-Encapsulated NiCo Alloy Enables High-Current Nitrate-to-Ammonia Electrosynthesis**

Qizhi Hu^1, †^, Siyu Zhu^2,^ ^†^, Biao Meng^1^, Mingdong Sun^1^, Mingzhen Wang^1^, Xiaoling Liu^1^, Shibo Xi^3^, Xiao Chi^4^, Xiaojiang Yu^5^, Jing Wu^1^, Yu Zhou^1,^ *, Yizhong Huang^2,^ *, and Jun Wang^1,^ *

^1^State Key Laboratory of Materials-Oriented Chemical Engineering, College of Chemical Engineering, Nanjing Tech University, Nanjing 211816, People’s Republic of China

^2^School of Materials Science and Engineering, Nanyang Technological University, 50 Nanyang Avenue, Singapore 639798, Singapore

^3^Institute of Sustainability for Chemicals, Energy and Environment (ISCE2), Agency for Science, Technology and Research (A*STAR), 1 Pesek Road Jurong Island, Singapore 627833, Singapore

^4^National University of Singapore, Department of Physics, Singapore 117576, Singapore

^5^National University of Singapore, Singapore Synchrotron Light Source, 5 Research Link, Singapore 117603, Singapore

† Qizhi Hu and Siyu Zhu have contributed equally to this work.

* Corresponding authors. E-mail: [njutzhouyu@njtech.edu.cn](mailto:njutzhouyu@njtech.edu.cn) (Yu Zhou); [yzhuang@ntu.edu.sg](mailto:yzhuang@ntu.edu.sg) (Yizhong Huang); [junwang@njtech.edu.cn](mailto:junwang@njtech.edu.cn) (Jun Wang)

**S1 Experimental Section**

***S1.1 Materials***

All chemicals are commercially available and used as received. Nickel acetate tetrahydrate (Ni(CH_3_COO)_2_·4H_2_O, 99%), cobalt nitrate hexahydrate (Co(NO_3_)_2_·6H_2_O, 99%), superconducting carbon black (K90), methanol (MeOH, 99.8%), ethanol (EtOH, 99.7%), chloroform (99%), potassium nitrite (97%), potassium nitrate (99%), and N,N-dimethylformamide (99%) was purchased from Aladdin Chemical. 2-methylimidazole (98%), terephthalic acid (99%), and potassium hydroxide (99%) were provided by Macklin Chemical. Nickel nitrate hexahydrate (Ni(NO_3_)_2_·6H_2_O, 98%), and ammonium fluoride (NH_4_F, 96%) was purchased from Sinopharm Chemical Reagent Co. Ltd. Deionized water was purchased from Watson’s. Nickel foam (NF) was obtained from Guangjiayuan New Material Co., Ltd. The H-type reactor, MEA reactor, Pt foil electrode, working electrode clip, and Ag/AgCl reference electrode were supplied by GaossUnion (Tianjin) Photoelectric Technology Co., Ltd. Nafion 211 and Nafion 117 proton-exchange membranes were purchased from E. I. du Pont de Nemours and Company. Carbon paper (TGP-H-060) was obtained from Toray Industries, Inc.

***S1.2 Characterization***

Powder X-ray diffraction (PXRD) patterns were collected on a Rigaku SmartLab diffractometer using nickel-filtered Cu *K*α radiation over a 2*θ* range of 5~80º (40 kV, 100 mA, 0.2º s^−1^). Scanning electron microscope (SEM) images were obtained on a Hitachi S-4800 field emission scanning electron microscope. Transmission electron microscopy (TEM) investigations were conducted using a JEOL microscope (Japan) with an accelerating voltage of 200 kV. Element analysis was conducted on a CHN elemental analyzer (FlashEA 1112). The metal contents were quantified by inductively coupled plasma optical emission spectrometry (ICP-OES). Nitrogen sorption experiments were carried out at 77 K on a BELSORP-MAX analyzer. The pore size distribution was calculated using the Nonlocal Density Functional Theory (NLDFT) with a cylindrical pore model. X-ray photoelectron spectroscopy (XPS) was performed on a Kratos AXIS Supra spectrometer equipped with Al *K*α radiation. Synchrotron X-ray absorption fine structure (XAFS) spectra at the Co *K*-edge and Ni *K*-edge were conducted in transmission mode at the XAFS for Catalysis and Materials Science (XAFCA) beamline of the Singapore Synchrotron Light Source (SSLS). Synchrotron-based soft X-ray absorption (XAS) spectra were recorded at the Surface, Interface, and Nanostructure Science (SINS) beamline of the SSLS, utilizing a Scienta R4000 electron energy analyzer. *In situ* Fourier-transform infrared spectroscopy (*in situ* FT-IR) measurements were performed on a Thermo Scientific Nicolet iS50 FT-IR spectrometer. Online differential electrochemical mass spectrometry (DEMS) measurements were performed using an in-situ electrochemical mass spectrometer (PM-DEMS, Shanghai Pro-Tech Limited Company) to monitor volatile intermediates and products during NO_3_^−^RR.

***S1.3 Catalyst preparation***

*Synthesis of* *NiCoS_x_/NF*

Nickel foam (NF, 2×2 cm^2^ pieces) was ultrasonically cleaned sequentially in 3 M HCl, water, and ethanol to remove surface impurities, followed by vacuum drying at 60 °C for 24 h. A precursor solution was prepared by dissolving Ni(NO_3_)_2_·6H_2_O (1 mmol), Co(NO_3_)_2_·6H_2_O (1 mmol), urea (7.5 mmol), and NH_4_F (5 mmol) in 30 mL H_2_O, followed by stirring at room temperature for 30 min. The cleaned NF was immersed in the solution and hydrothermally treated at 120 °C for 10 h. The resulting samples were washed, dried, and designated as NiCoLDH/NF. Subsequently, NiCoLDH/NF was sulfurized by reacting with 20 mL of 50 M Na_2_S·9H_2_O solution at 100 °C for 2 h. After washing, ethanol-assisted sonication, and drying, the intermediate product was obtained and denoted as NiCoS*ₓ*-Pre/NF. Finally, the material was annealed at 300 °C for 0.5 h under N_2_ flow to yield the target NiCoS*ₓ*/NF.

***S1.4*** *Product detection and quantification*

*Ammonia quantification*

The concentration of NH_3_ was determined using the indophenol blue spectrophotometric method. After electrolysis, 0.5 mL of electrolyte was withdrawn and diluted to fall within the detection range. Subsequently, 5 mL of the diluted solution was then mixed with 2 mL of 1 M NaOH solution containing 5 wt% salicylic acid and 5 wt% sodium citrate, 1 mL of 0.05 M NaClO solution, and 0.2 mL of 1.0 wt% sodium nitroferricyanide solution. The mixture was allowed to stand at room temperature for 2 h to ensure complete color development. The absorbance at 655 nm was measured using a UV–vis spectrophotometer (Shimadzu UV-2600). Standard calibration curves were established with a series of NH₄Cl solutions to determine the NH_3_ concentration.

*Nitrite quantification*

The concentration of NO_2_^−^ was quantified by the Griess colorimetric method. The detection reagent was prepared by dissolving sulfanilamide (4 g), phosphoric acid (10 mL), and N-(1-naphthyl)ethylenediamine dihydrochloride (0.2 g) in water (50 mL). After electrolysis, 0.5 mL of electrolyte was collected and diluted to fall within the detection range. Then, 5 mL of the diluted solution was then mixed with 0.1 mL of the detection reagent. After standing for 20 min at room temperature, the absorbance was measured at 540 nm on a UV-vis spectrophotometer (Shimadzu UV-2600). Standard calibration curve was established with a series of standard sodium nitrite solutions to quantify the NO_2_^−^ concentration.

*Nitrate detection*

The concentration of nitrate was determined by UV-vis spectrophotometry. Typically, 4 mL of the diluted electrolyte was mixed with 1 mL of 1.0 M HCl and 0.1 mL of sulfamic acid solution (0.8 wt.%). After standing at room temperature for 20 min, the absorbance values at 220 and 275 nm were recorded using a UV-vis spectrophotometer. The corrected absorbance was calculated according to (A = A_220nm_−2A_275nm_). A calibration curve was obtained by linear fitting of standard potassium nitrate solutions with known concentrations, and the nitrate concentration in the electrolyte was then determined from the corrected absorbance and the corresponding calibration curve.

*Formate* *quantification*

The concentration of formate was determined by high-performance liquid chromatography (HPLC). After electrocatalytic methanol oxidation, 1 mL of the anolyte was collected and mixed with 1 mL of 2 M HCl. The resulting solution was analyzed on an HPLC system (Thermo Scientific^TM^ UltiMate^TM^ 3000) equipped with a diode array detector (DAD, detection wavelength: 210 nm) and a Shodex SUGAR SH1011 column. The mobile phase was 5 mM H_2_SO_4_ (0.7 mL min^−1^) with the column temperature of 80 °C.

*Hydrogen and nitrogen quantification*

After the reaction, gas samples were collected using a gas-tight syringe and analyzed by GC (GC-9860-5CNJ) equipped with FID and TCD, using a 5 Å molecular sieve column (2 m, 60 °C) with Ar as the carrier gas.

*S1.5 ^15^N isotope-labeling experiment*

To verify the nitrogen source of the produced NH_3_, an isotope-labeling experiment was carried out using an electrolyte composed of 1.0 M KOH and 0.1 M K^15^NO_3_ (98 atom% ^15^N). Electroreduction was performed at −0.6 V vs. RHE for 1 h, after which the generated ^15^NH_4_^+^ was analyzed by ^1^H nuclear magnetic resonance (^1^H NMR). The detection procedure for ^15^NH_4_^+^ was identical to that used for ^14^NH_4_^+^.

*S1.6* ***Online differential electrochemical mass spectrometry measurements***

The measurements were performed on an in-situ electrochemical mass spectrometer (PM-DEMS, Shanghai Pro-Tech Limited Company). The electrolyte (1 M KOH + 0.1 M KNO_3_) was continuously introduced into the electrochemical cell using a peristaltic pump. NiCo@NC, Pt foil, and Hg/HgO were used as the working, counter, and reference electrodes, respectively. After the mass-signal baseline became stable, chronoamperometric measurements were conducted at −0.5 V_RHE_, and the corresponding mass signals were recorded. Each test was repeated for five cycles under identical conditions after the signals returned to baseline to minimize accidental errors.

*S1.7 Computational Methods*

All density functional theory (DFT) calculations were performed with spin polarization using the Vienna Ab initio Simulation Package (VASP). The projector augmented-wave (PAW) method was applied with a plane-wave basis set to describe the interaction between valence electrons and ionic cores. Exchange-correlation effects were treated using the Perdew-Burke-Ernzerhof (PBE) functional within the generalized gradient approximation (GGA). A kinetic energy cutoff of 400 eV was used to ensure reliable convergence. Grimme’s DFT-D3(BJ) dispersion correction was included to account for van der Waals (vdW) interactions. A vacuum layer of at least 15 Å was introduced along the z-direction to avoid artificial interactions between periodic slabs.

A periodic core-shell model was constructed to represent NiCo alloy nanoparticles encapsulated by an N-doped graphitic carbon shell (NiCo@NC). First, a bulk fcc NiCo alloy with a Ni/Co atomic ratio of 1/8 was optimized, and then a two-layer NiCo (111) slab with a 4 × 4 surface cell was cleaved as the alloy core. An N-doped graphene layer was placed on the NiCo (111) surface to mimic the carbon shell, and its orientation was adjusted to reduce lattice mismatch with the alloy surface. Pyridinic-N and graphitic-N configurations were introduced into the graphitic carbon shell near the metal-carbon interface to emulate the experimentally observed N-doped carbon environment. For comparison, analogous Ni@NC and Co@NC models were built by replacing the NiCo alloy slab with single-component Ni or Co slabs while maintaining the same N-doped carbon-shell structure. During geometry optimization, the N-doped carbon shell and surface metal layers were fully relaxed.

To determine the representative adsorption site in NiCo@NC, NO_3_^−^, NO_2_^−^, and NO adsorption was examined at pyridinic-N, graphitic-N, and graphitic-C sites on the N-doped graphitic shell. Among these sites, the pyridinic-N-adjacent configuration exhibited the strongest adsorption toward the nitrogen-containing reactants and intermediates, indicating that pyridinic-N provides a more favorable local electronic environment for NO_3_^−^RR. Therefore, pyridinic-N-adjacent sites were selected as the representative adsorption sites for subsequent free-energy calculations. Charge redistribution at the metal-carbon interface was analyzed using charge density difference maps and Bader charge analysis.

Gibbs free energy profiles for NO_3_^−^ reduction reaction (NO_3_^−^RR), hydrogen evolution reaction (HER), and water dissociation were calculated based on the optimized adsorption configurations. No explicit solvent molecules were included in the present calculations, and no additional external electric field, applied potential, or potential-dependent correction was introduced. For proton–electron transfer steps, the computational hydrogen electrode (CHE) model was used, in which the chemical potential of one proton–electron pair was referenced to half of a hydrogen molecule under standard conditions. Unless otherwise stated, the reported free energies correspond to U = 0 V_RHE_ without additional potential correction.

The adsorption energy (*E*ₐ) of a chemical species *A* on the catalyst surface was calculated as:

*E*_a_=*E*_*A_−*E*_*_−*E*_A_

where 𝐸_*A_ represents the total energy of reactant A adsorbed on the catalyst surface, 𝐸_*_ is the energy of the bare catalyst surface, and *E*_A_ is the energy of reactant A in the gas phase.

**S2 Supplementary Figures**

**Fig. S1** PXRD patterns of Ni-ZB, Co-ZB, and NiCo-ZB.

**Fig. S2** PXRD patterns of Ni@NC, Co@NC, and NiCo@NC.

**Fig. S3** Magnified XRD patterns of NiCo@NC, Ni@NC, and Co@NC in the 2*θ* range of **a** 43-46°, **b** 50-53°, and **c** 74-78°.

**Fig. S4** Nitrogen sorption isotherms Ni@NC, Co@NC, and NiCo@NC.

**Fig. S5** Pore size distribution curves of Ni@NC, Co@NC, and NiCo@NC.

**Fig. S6** SEM images of **a**,**b** Co@NC, **c**,**d** Ni@NC, **e**,**f** NiCo@NC, and **g**,**h** spent NiCo@NC.

**Fig. S7 a** TEM image and **b** particle size distribution curve of NiCo@NC. **c** TEM image and **d** particle size distribution curve of Ni@NC. **e** TEM image and **f** particle size distribution curve of Co@NC.

**Fig. S8** High resolution TEM images of **a**,**b** NiCo@NC, **c**,**d** Ni@NC, and **e**,**f** Co@NC.

**Fig. S9** Elemental mapping images of C, Co, and Ni for NiCo@NC.

**Fig. S10** Raman spectra of Co@NC, Ni@NC, and NiCo@NC.

**Fig. S11** Survey scan XPS spectra of Co@NC, Ni@NC, and NiCo@NC.

**Fig. S12** High-resolution C 1s XPS spectra of Co@NC, Ni@NC, and NiCo@NC.

**Fig. S13** High-resolution N 1s XPS spectra of Co@NC, Ni@NC, and NiCo@NC.

**Fig. S14** High-resolution Ni 2p XPS spectra of Co@NC, Ni@NC, and NiCo@NC.

**Fig. S15** High-resolution Co 2p XPS spectra of Co@NC, Ni@NC, and NiCo@NC.

**Fig. S16** FT-EXAFS curves for the experimental and fitted profiles at the Co *K*-edge of **a** Co foil, **b** CoO, **c** NiCo@NC, and **d** Co@NC.

**Fig. S17** FT-EXAFS curves for the experimental and fitted profiles at the Ni *K*-edge of **a** Ni foil, **b** NiO, **c** NiCo@NC, and **d** Ni@NC.

**Fig.** **S18** UV-vis spectra and corresponding calibration curves for **a**,**b** NH_3_ quantification *via* the indophenol blue method and **c**,**d** NO_2_**^−^** determination *via* the Griess reagent method. **e**,**f** UV-vis spectra and calibration curve for NO_3_**^−^** quantification. **g** Linear GC calibration curves of H_2_. **h** Linear GC calibration curves of N_2_. **i** HPLC calibration curves of formate. The corresponding calibration equations and limits of detection are summarized in Table S8.

**Fig. S19** Electrochemical NO_3_^−^RR performance of NiCo@NC, Co@NC, and Ni@NC in an H-type cell. **a** NH_3_ FE and yield rate over different catalysts. Influence of **b** applied potential, **c** nitrate concentration, and **d** pyrolysis temperature on the NO_3_^−^RR performance of NiCo@NC. Unless otherwise specified, the reaction conditions were: catalyst loading, 0.1 mg; electrolysis time, 1 h; electrolyte, 1 M KOH containing 0.1 M KNO_3_; electrode area, 1 cm^2^; applied potential, −0.6 V_RHE_.

**Fig. S20 a** TEM image and **b** particle size distribution curve of NiCo@NC-NB.

**Fig. S21** **a** LSV curves of NiCo@NC and NiCo@NC-NB in 1 M KOH with (solid lines) and without (dotted line) 0.1 M KNO_3_. **b** FE and productivity of NH_3_ over NiCo@NC and NiCo@NC-NB.

**Fig. S22** LSV curves of NiCo@NC measured in KNO_3_ electrolyte solution with different concentrations.

**Fig. S23** Effect of nitrate concentration on NO_3_^−^RR performance over NiCo@NC under neutral conditions. **a** LSV curves recorded in neutral electrolytes, **b** NH_3_ yield rates and **c** product Faradaic efficiencies as a function of NO_3_^−^ concentration. Reaction conditions: catalyst loading, 0.1 mg; electrolysis time, 1 h; electrolyte, 0.5 M Na_2_SO_4_, 0.1 M Na_2_CO_3_ containing different concentrations of NaNO_3_; electrode area, 1 cm^2^; applied potential, −0.8 V_RHE_.

**Fig. S24** ^1^H NMR spectra of the electrolytes after ^15^NO_3_^−^ and ^14^NO_3_^−^ electroreduction.

**Fig. S25** Nitrate conversion and yields of NH_3_, NO_2_^−^ and N_2_ over NiCo@NC during NO_3_^−^RR. Reaction conditions: catalyst, NiCo@NC; catalyst loading, 0.1 mg; electrolysis time, 1 h; electrolyte, 1 M KOH containing 0.1 M KNO_3_; applied potential, −0.6 V_RHE_.

**Fig. S26** Tafel slopes for NO_3_^−^RR, obtained from the kinetic region of the LSV curves.

**Fig. S27** CV curves of **a** NiCo@NC, **b** Co@NC, and **c** Ni@NC toward NO_3_^−^RR. **d** Linear plots of the oxidation peak current versus scan rate for the corresponding catalysts.

**Fig. S28 a** TEM image and **b** particle size distribution curve of spent NiCo@NC.

**Fig. S29** PXRD patterns of fresh NiCo@NC and spent NiCo@NC.

**Fig. S30** Cycling tests of Co@NC in NO_3_^−^RR. Reaction conditions: 0.1 mg Co@NC, −0.6 V_RHE_, 1 h, 1 M KOH, 0.1 M KNO_3_.

**Fig. S31** Schematic illustration of the ammonia-related product synthesis pathway showing the conversion of nitrate-containing influent through electrochemical NO_3_^−^RR to intermediate species, followed by capture and crystallization into NH_4_Cl.

**Fig. S32** FE and yield rate of NH_3_ and HCOOH in the NO_3_^−^RR||MORR system. ^a^Reaction conditions: NiCo@NC (1 mg), carbon paper (1 cm^2^), 1 M KOH, 1 M KNO_3_, H_2_O (200 mL) in cathodic electrolytic cell; NiCo-LDH (1 cm^2^), 1 M KOH, 1 M MeOH, H_2_O (200 mL) in anode electrolytic cell; 0.5 h.

**Fig. S33** Benchmark comparison of NH_3_ Faradaic efficiency and current density for NiCo@NC with recently reported MEA-based NO_3_^−^RR catalysts.

**Fig. S34** *In situ* FT-IR spectra of Ni@NC at different applied potentials.

**Fig. S35** *In situ* FT-IR spectra of Co@NC at different applied potentials.

**Fig. S36** *In situ* DEMS profiles NO_3_^−^RR over NiCo@NC.

**Fig. S37** Co *K*-edge FT-EXAFS curves for the experimental and fitted profiles of Co@NC in 1 M KOH with 0.1 M KNO_3_ at **a** OCP, **b** 0.1 V, **c** −0.1 V, and **d** after reaction.

**Fig. S38** Co *K*-edge FT-EXAFS curves for the experimental and fitted profiles of NiCo@NC in 1 M KOH with 0.1 M KNO_3_ at **a** OCP, **b** 0.1 V, **c** −0.1 V, and **d** after reaction.

**Fig. S39** Density of states and *d*-band centers of **a** Ni in NiCo@NC and Ni@NC, and **b** Co in NiCo@NC and Co@NC.

**Fig. S40** Charge density differences in **a** NiCo@NC, **b** Co@NC, and **c** Ni@NC interfaces. Yellow and cyan regions represent the accumulation and deficiency of electrons, respectively.

**Fig. S41** Schematic of electron transfer between NiCo NPs and N**_p_**–C**_g_** structure.

**Fig. S42** Adsorption energy of NO_3_^−^ at pyridinic-N, graphitic-C, and graphitic-N sites in NiCo@NC.

**Fig. S43** Adsorption energy of NO_2_^−^ at pyridinic-N, graphitic-C, and graphitic-N sites in NiCo@NC.

**Fig. S44** Adsorption energy of NO at pyridinic-N, graphitic-C, and graphitic-N sites in NiCo@NC.

**Fig. S45** Optimized adsorption configurations of different intermediates for NO_3_^−^RR on Co@NC.

**Fig. S46** Optimized adsorption configurations of different intermediates for NO_3_^−^RR on NiCo@NC.

**Fig. S47** Optimized adsorption configurations of different intermediates for NO_3_^−^RR on Ni@NC.

**Fig. S48** LSV curves of NiCo@NC, Co@NC, and Ni@NC in 1 M KOH electrolyte with and without 0.1 M KNO_2_.

**Fig. S49** Adsorption energy of NO on NiCo@NC, Ni@NC, and Co@NC.

**Fig. S50** Adsorption energy of NO_3_^−^ on NiCo, Ni, Co, and carbon-layer.

**Fig. S51** Adsorption energy of NO_2_^−^ on NiCo, Ni, Co, and carbon-layer.

**Fig. S52** Adsorption energy of NO on NiCo, Co, Ni, and carbon-layer.

**Fig. S53** Gibbs free energy profiles of water dissociation over NiCo, Co, Ni, and carbon-layer.

**S3 Supplementary Tables**

**Table S1** Textural properties

| Entry | Sample | S_BET_^a^ (m^2^/g) | V_p_^b^ (cm^3^/g) | D_p_^c^ (nm) |
| --- | --- | --- | --- | --- |
| 1 | NiCo@NC | 186 | 0.34 | 1.2 |
| 2 | Ni@NC | 177 | 0.48 | 0.9 |
| 3 | Co@NC | 152 | 0.36 | 1.6 |

^a^ BET surface area, ^b^ Total pore volume, ^c^ Average pore diameter.

**Table S2** Elemental composition

| Entry | Sample | C (wt%)^a^ | N (wt%)^a^ | H (wt%)^a^ | Co (wt%)^b^ | Ni (wt%)^b^ |
| --- | --- | --- | --- | --- | --- | --- |
| 1 | NiCo@NC | 59.9 | 0.4 | 2.4 | 28.9 | 2.7 |
| 2 | Co@NC | 63.3 | 0.5 | 2.0 | 26.3 | - |
| 3 | Ni@NC | 58.1 | 0.9 | 1.9 | - | 27.1 |

^a^Measured by element analyses, ^b^Measured by ICP-OES.

**Table S3** Molar ratios of N, Ni, and Co in different catalysts.

| Sample | N : Ni: Co |
| --- | --- |
| NiCo@NC | 1 : 1.61: 17.17 |
| Co@NC | 1 : 0 : 12.5 |
| Ni@NC | 1 : 7.19 : 0 |

**Table S4** EXAFS fitting results at the Co *K*-edge of different samples

| Sample | Shell | CN | R (Å) | σ^2^ (Å^2^) | R-factor* |
| --- | --- | --- | --- | --- | --- |
| Co foil | Co–Co | 12.0 | 2.49 | 0.0055 | 0.0017 |
| CoO | Co–Co | 12.0 | 2.99 | 0.0096 | 0.0065 |
|  | Co–O | 6.0 | 2.06 | 0.0230 |  |
| NiCo@NC | Co–Co(Ni) | 10.9 | 2.52 | 0.0071 | 0.0033 |
| Co@NC | Co–Co | 10.1 | 2.52 | 0.0074 | 0.0037 |

CN, coordination number; R, bonding distance; σ^2^, Debye-Waller factor.

**Table S5** EXAFS fitting results at the Ni *K*-edge of different samples

| Sample | Shell | CN | R (Å) | σ^2^ (Å^2^) | R-factor* |
| --- | --- | --- | --- | --- | --- |
| Ni foil | Ni–Ni | 12 | 2.4813 | 0.00518 | 0.0074 |
| NiO | Ni–Ni | 12 | 2.9434 | 0.00626 | 0.0037 |
|  | Ni–O | 6 | 2.0872 | 0.00577 |  |
| NiCo@NC | Ni–Ni(Co) | 11.2 | 2.4780 | 0.00564 | 0.0067 |
| Ni@NC | Ni–Ni | 11.8 | 2.4802 | 0.00570 | 0.0043 |

CN, coordination number; R, bonding distance; σ^2^, Debye-Waller factor.

**Table S6** Calibration equations and limits of detection for product quantification.

| Analyte | Calibration equation | R^2^ | Limits of detection |
| --- | --- | --- | --- |
| NH_3_ | y=0.0586x+0.0026 | 0.999 | 0.02 μg mL^−1^ |
| NO_2_^−^ | y=0.1228x+0.0006 | 0.999 | 0.05 μg mL^−1^ |
| H_2_ | y=0.0939x | 0.999 | 0.03 μg mL^−1^ |
| N_2_ | y=0.2134x | 0.999 | 0.09 μg mL^−1^ |
| NO_3_^−^ | y=0.198x+0.0052 | 0.999 | 0.05 μg mL^−1^ |
| HCOOH | y=0.8136x | 0.999 | 0.08 μmmol mL^−1^ |

**Table S7** Comparison of NO_3_^−^RR performance in MEA reactor

| Catalysts | Electrolyte | Current  (A cm^−2^) | FE  (%) | Voltage  (V) | Ref. |
| --- | --- | --- | --- | --- | --- |
| a/c-Cu | 1 M KOH,  0.2 M KNO_3_ | 3.5 | 83 | 2.6 | [S1] |
|  |  | 1.5 | 90 | 2.0 |  |
| Ag_1_@Cu_2_O | 1 M KOH,  0.1 M KNO_3_ | 0.6 | 90 | 1.8 | [S2] |
| CuCoNiRuPt | 1 M KOH,  0.5 M KNO_3_ | 1.0 | 92 | 2.2 | [S3] |
| (Cu_7_/Ag_3_)_7_-Ru_3_/C | 1 M KOH,  0.1 M KNO_3_ | 1.8 | 90 | 2.0 | [S4] |
| Co@Ga LMMSs | 1 M NaOH,  1 M NaNO_3_ | 1.0 | 90 | 2.2 | [S5] |
| Co_6_Ni_4_ | 1 M KOH,  0.3 M KNO_3_ | 0.35 | 98 | 2.2 | [S6] |
| Co_9_Cu@CC | 1 M KOH,  0.1 M KNO_3_ | 0.4 | 80 | 2.0 | [S7] |
| Ru_1_@(Cu,Ni)_2_O_1-δ_ | 1 M KOH,  0.1 M KNO_3_ | 0.4 | 95 | 1.5 | [S8] |
| AgRu-15 | 1 M KOH,  0.1 M KNO_3_ | 2.0 | 81 | 0.6 | [S9] |
| NiCo@NC | 1 M KOH,  1 M KNO_3_ | 2.1 | 90 | 2.8 | This work |

**Table S8** EXAFS fitting results at the Co *K*-edge of Co@NC at selected potentials in 1 M KOH and 0.1 M KNO_3_

| Sample | Shell | CN | R (Å) | σ^2^ (Å^2^) | R-factor* |
| --- | --- | --- | --- | --- | --- |
| OCP | Co–Co | 10.1 | 2.55 | 0.0054 | 0.0062 |
| 0.1 V | Co–Co | 9.8 | 2.58 | 0.0042 | 0.0198 |
| −0.1 V | Co–Co | 9.7 | 2.59 | 0.0034 | 0.0013 |
| After reaction | Co–Co | 9.8 | 2.58 | 0.0035 | 0.0158 |

CN, coordination number; R, bonding distance; σ^2^, Debye-Waller factor.

**Table S9** EXAFS fitting results at the Co *K*-edge of NiCo@NC at selected potentials in 1 M KOH and 0.1 M KNO_3_

| Sample | Shell | CN | R (Å) | σ^2^ (Å^2^) | R-factor* |
| --- | --- | --- | --- | --- | --- |
| OCP | Co–Co | 10.3 | 2.53 | 0.0058 | 0.0066 |
| 0.1 V | Co–Co | 10.1 | 2.55 | 0.0060 | 0.0053 |
| −0.1 V | Co–Co | 10.0 | 2.55 | 0.0058 | 0.0065 |
| After reaction | Co–Co | 10.3 | 2.53 | 0.0058 | 0.0063 |

CN, coordination number; R, bonding distance; σ^2^, Debye-Waller factor.

**Table S10** Comparison of NO_3_^−^RR performance in H-type cell

| Catalysts | Year | Electrolyte | Potential  (V *vs.* RHE) | FE  (%) | Yield rate  (mol h^−1^ g_cat_^−1^) | Ref. |
| --- | --- | --- | --- | --- | --- | --- |
| Fe/Cu | 2023 | 1 M KOH,  0.1 M KNO_3_ | −0.5 | 93 | 1.08 | [S10] |
| BCN@Cu/CNT | 2022 | 1 M KOH,  0.1 M KNO_3_ | −0.6 | 95 | 10.13 | [S11] |
| CuPd | 2023 | 1 M KOH,  0.1 M KNO_3_ | −0.9 | 95 | 1.06 | [S12] |
| CoNi-NC-400 | 2023 | 1 M KOH,  0.1 M KNO_3_ | −0.6 | 60 | 1.25 | [S13] |
| CuCo_2_O_4_/CFs | 2022 | 1 M KOH,  0.1 M KNO_3_ | −0.3 | 82 | 0.39 | [S14] |
| CuCo | 2022 | 1 M KOH,  0.1 M KNO_3_ | −0.2 | 95 | 0.96 | [S15] |
| Ag/ZnO | 2022 | 1 M KOH,  0.1 M KNO_3_ | −0.6 | 66 | 0.52 | [S16] |
| Cu-NBs-100 | 2023 | 1 M KOH,  0.1 M KNO_3_ | −0.15 | 95 | 0.65 | [S17] |
| MoO_x_/C | 2024 | 1 M KOH,  0.1 M KNO_3_ | −0.5 | 98 | 5.39 | [S18] |
| PdCu metallene | 2025 | 0.5 M K_2_SO_4_,  0.1 M KNO_3_ | −1.2 | 91 | 17.35 | [S19] |
| Cu-SNC-2 | 2025 | 1 M KOH,  0.1 M KNO_3_ | −0.3 | 92 | 1.18 | [S20] |
| Cu_2_O/Cu(OH)_2_ @Ni(OH)_2_ | 2026 | 1 M KOH,  0.1 M KNO_3_ | −0.6 | 99 | 1.14 | [S21] |
| Co_2_/NSC | 2026 | 1 M KOH,  0.1 M KNO_3_ | −0.4 | 92 | 5.95 | [S22] |
| Sb_2_W_18_Fe_4_ | 2026 | 0.5 M K_2_SO_4_, 0.1 M KNO_3_ | −0.99 | 93 | 2.06 | [S23] |
| Cu_8_@Cu_6_-PPh_3_ | 2026 | 1 M KOH,  0.1 M KNO_3_ | −0.7 | 97 | 2.20 | [S24] |
| Cu-Co_3_S_4_ | 2026 | 1 M KOH,  0.1 M KNO_3_ | −0.8 | 95 | 5.56 | [S25] |
| NiCo@NC | 2026 | 1 M KOH,  0.1 M KNO_3_ | −0.6 | 93 | 6.95 | This  work |
|  |  | 1 M KOH,  1 M KNO_3_ | −0.6 | 95 | 187 |  |

**Supplementary References**

[S1]	Y. Wang, S. Wang, Y. Fu, J. Sang, P. Wei et al., Ammonia electrosynthesis from nitrate using a stable amorphous/crystalline dual-phase Cu catalyst. Nat. Commun. **16**(1), 897 (2025). <https://doi.org/10.1038/s41467-025-55889-9>

[S2]	L. Zhang, Y. Cai, Y. Li, C. Sun, Y. Xiao et al., Unlocking high-current-density nitrate reduction and formaldehyde oxidation synergy for scalable ammonia production and fixation. Energy Environ. Sci. **18**(6), 2804-2816 (2025). <https://doi.org/10.1039/d4ee04382k>

[S3]	S. Li, Y. Su, H. Wan, Z. Jiang, T. Niu et al., A Reaction-diffusion-coupled strategy for ampere-level electrocatalytic nitrate reduction to ammonia. Nano Lett. **25**(38), 14185-14194 (2025). <https://doi.org/10.1021/acs.nanolett.5c03905>

[S4]	R. Jia, X. Zhang, L. Gan, M. Tahir, Z.-F. Huang et al., Boosting electrocatalytic nitrate reduction to ammonia with a Cu/Ag-Ru tandem catalyst at industrial-scale current density. J. Mater. Chem. A **13**(8), 5732-5743 (2025). <https://doi.org/10.1039/d4ta08066a>

[S5]	W. Chen, W. Zeng, Z. Chen, Y. Zou, C. Zhang et al., Liquid metal dynamic interface enabled reverse hydrogen spillover boosting electrocatalytic nitrate reduction. Angew. Chem., Int. Ed. **65**(4), e202516997 (2026). <https://doi.org/10.1002/anie.202516997>

[S6]	X. Shi, W.-H. Huang, J. Rong, M. Xie, Q. Wa et al., Revealing and modulating catalyst reconstruction for highly efficient electrosynthesis of ammonia. Nat. Commun. **16**(1), 6161 (2025). <https://doi.org/10.1038/s41467-025-61075-8>

[S7]	Y. Wan, Y. Chen, Y. Tang, W. Yan, J. Zhang et al., Stepwise upgrading of waste nitrate by fast joule‐heating and electrochemical reduction at wide potentials. Adv. Funct. Mater. **1**(1), e76112 (2026). <https://doi.org/10.1002/adfm.76112>

[S8]	S. Zhang, C. Liu, J. Jiang, W. Liu, Z. Lin et al., Expanding two‐dimensional electrochemical window enables durable copper‐based electrocatalysts for nitrate‐to‐ammonia conversion. Angew. Chem., Int. Ed. **65**(4), e22700 (2026). <https://doi.org/10.1002/anie.202522700>

[S9]	L. Zhang, R. Liu, X. Liang, H. Gao, Y. Fan et al., Exploiting underpotential deposited hydrogen enables energy-efficient nitrate electroreduction to ammonia. Nat. Commun. **17**(1), 4652 (2026). <https://doi.org/10.1038/s41467-026-71299-x>

[S10]	S. Zhang, J. Wu, M. Zheng, X. Jin, Z. Shen et al., Fe/Cu diatomic catalysts for electrochemical nitrate reduction to ammonia. Nat. Commun. **14**(1), 3634 (2023). <https://doi.org/10.1038/s41467-023-39366-9>

[S11]	X. Zhao, X. Li, H. Zhang, X. Chen, J. Xu et al., Atomic-dispersed copper simultaneously achieve high-efficiency removal and high-value-added conversion to ammonia of nitrate in sewage. J. Hazard. Mater. **424**(4), 127319 (2022). <https://doi.org/10.1016/j.jhazmat.2021.127319>

[S12]	Z. Song, L. Qin, Y. Liu, Y. Zhong, Q. Guo et al., Efficient electroreduction of nitrate to ammonia with CuPd nanoalloy catalysts. ChemSusChem **16**(22), e202300202 (2023). <https://doi.org/10.1002/cssc.202300202>

[S13]	F. Lei, Y. Zhang, M. Xu, K. Li, M. Zhang et al., Energy-efficient ammonia synthesis from nitrate via CoNi alloys incorporated in carbon frameworks. ACS Sustainable Chem. Eng. **11**(24), 9057-9064 (2023). <https://doi.org/10.1021/acssuschemeng.3c01688>

[S14]	Z. Niu, S. Fan, X. Li, P. Wang, Z. Liu et al., Bifunctional copper-cobalt spinel electrocatalysts for efficient tandem-like nitrate reduction to ammonia. Chem. Eng. J. **450**, 138343 (2022). <https://doi.org/10.1016/j.cej.2022.138343>

[S15]	J. Y. Fang, Q. Z. Zheng, Y. Y. Lou, K. M. Zhao, S. N. Hu et al., Ampere-level current density ammonia electrochemical synthesis using CuCo nanosheets simulating nitrite reductase bifunctional nature. Nat. Commun. **13**(1), 7899 (2022). <https://doi.org/10.1038/s41467-022-35533-6>

[S16]	F. Lei, K. Li, M. Yang, J. Yu, M. Xu et al., Electrochemical reduction of nitrate on silver surface and an in situ Raman spectroscopy study. Inorg. Chem. Front. **9**(11), 2734-2740 (2022). <https://doi.org/10.1039/d2qi00489e>

[S17]	Q. Hu, Y. Qin, X. Wang, Z. Wang, X. Huang et al., Reaction intermediate-mediated electrocatalyst synthesis favors specified facet and defect exposure for efficient nitrate-ammonia conversion. Energy Environ. Sci. **14**(9), 4989-4997 (2021). <https://doi.org/10.1039/d1ee01731d>

[S18]	M. Sun, G. Wu, J. Jiang, Y. Yang, A. Du et al., Carbon‐anchored molybdenum oxide nanoclusters as efficient catalysts for the electrosynthesis of ammonia and urea. Angew. Chem., Int. Ed. **62**(19), e202301957 (2023). <https://doi.org/10.1002/anie.202301957>

[S19]	Y. Zhou, L. Zhang, Z. Zhu, M. Wang, N. Li et al., Optimizing intermediate adsorption over PdM (M = Fe, Co, Ni, Cu) bimetallene for boosted nitrate electroreduction to ammonia. Angew. Chem., Int. Ed. **63**(18), e202319029 (2024). <https://doi.org/10.1002/anie.202319029>

[S20]	M. Liu, J. Qi, G. Luo, Y. Cui, W. Wei et al., Asymmetric N, S‐coordinated Cu single‐atom catalysts for high‐performance electrocatalytic nitrate‐to‐ammonia. Adv. Funct. Mater. **36**(25), e23666 (2025). <https://doi.org/10.1002/adfm.202523666>

[S21]	T. Lv, L. Yang, C. Hong, Y. Zhu, J. Shen et al., Electronic interaction at Cu–O–Ni heterointerface promotes electrocatalytic nitrate reduction to ammonia and zinc‐nitrate battery. Adv. Sci. **13**(14), e21252 (2026). <https://doi.org/10.1002/advs.202521252>

[S22]	J. Wang, X. Kang, Z. Chu, P. Wu, Z. Chen et al., Machine learning driven high‐throughput screening of asymmetric dinuclear cobalt for nitrate‐to‐ammonia reduction with near‐100% selectivity. Adv. Energy Mater. **16**(11), e06009 (2026). <https://doi.org/10.1002/aenm.202506009>

[S23]	W.-J. Xia, Z.-C. Liu, J.-H. Huang, J.-B. Yang, Y.-Q. Sun et al., Boosting active hydrogen kinetics of electrocatalytic nitrate-to-ammonia via tuning electron structure of polyoxometalates. Appl. Catal., B **387**(126472 (2026). <https://doi.org/10.1016/j.apcatb.2026.126472>

[S24]	C. Li, J. Jia, Y. Fang, S. Jin, Y. Du et al., Highly efficient nitrate to ammonia electrocatalysis enabled by inverse core-shell isostructural Cu_14_ Nanoclusters. Adv. Funct. Mater. **36**(37), 74425 (2026). <https://doi.org/10.1002/adfm.74425>

[S25]	R. Li, R. Ma, L. L. Zhang, M. Jiao, Z. Zhou, Sulfur‐bridge engineering enables reverse hydrogen spillover to atomic Cu for nitrate‐to‐ammonia electrocatalysis. Angew. Chem., Int. Ed. **65**(18), e7688164 (2026). <https://doi.org/10.1002/anie.7688164>
